# Regulation of PKD by the MAPK p38δ in Insulin Secretion and Glucose Homeostasis

**DOI:** 10.1016/j.cell.2008.11.018

**Published:** 2009-01-23

**Authors:** Grzegorz Sumara, Ivan Formentini, Stephan Collins, Izabela Sumara, Renata Windak, Bernd Bodenmiller, Reshma Ramracheya, Dorothée Caille, Huiping Jiang, Kenneth A. Platt, Paolo Meda, Rudolf Aebersold, Patrik Rorsman, Romeo Ricci

**Affiliations:** 1 Institute of Cell Biology, ETH Zurich, CH-8093 Zurich, Switzerland; 2 Oxford Centre for Diabetes, Endocrinology and Metabolism, University of Oxford, Oxford OX3 7LJ, United Kingdom; 3 Institute of Biochemistry, ETH Zurich, CH-8093 Zurich, Switzerland; 4 Institute of Molecular Systems Biology, ETH Zurich, CH-8093 Zurich, Switzerland; 5 Faculty of Science, University of Zurich, CH-8006 Zurich, Switzerland; 6 Department of Cell Physiology and Metabolism, University of Geneva, CH-1211 Geneva, Switzerland; 7 Department of Biotherapeutics and Integrative Biology, Boehringer Ingelheim Pharmaceuticals, Inc., Ridgefield, Connecticut, CT 06877, USA; 8 Lexicon Pharmaceuticals, The Woodlands, Texas, TX 77381, USA; 9 Institute for Systems Physiology, Seattle, WA 98103, USA; 10 Competence Center for Systems Physiology and Metabolic Diseases, CH-8093 Zurich, Switzerland

**Keywords:** SIGNALING, CELLBIO, HUMDISEASE

## Abstract

Dysfunction and loss of insulin-producing pancreatic β cells represent hallmarks of diabetes mellitus. Here, we show that mice lacking the mitogen-activated protein kinase (MAPK) *p38δ* display improved glucose tolerance due to enhanced insulin secretion from pancreatic β cells. Deletion of *p38δ* results in pronounced activation of protein kinase D (PKD), the latter of which we have identified as a pivotal regulator of stimulated insulin exocytosis. p38δ catalyzes an inhibitory phosphorylation of PKD1, thereby attenuating stimulated insulin secretion. In addition, *p38δ* null mice are protected against high-fat-feeding-induced insulin resistance and oxidative stress-mediated β cell failure. Inhibition of PKD1 reverses enhanced insulin secretion from *p38δ*-deficient islets and glucose tolerance in *p38δ* null mice as well as their susceptibility to oxidative stress. In conclusion, the p38δ-PKD pathway integrates regulation of the insulin secretory capacity and survival of pancreatic β cells, pointing to a pivotal role for this pathway in the development of overt diabetes mellitus.

## Introduction

Diabetes results from insufficient (absolute or relative) insulin secretion. In type 1 diabetes, the insulin-producing β cells are destroyed by an autoimmune attack (von Herrath et al., 2007). Type 2 diabetes is often linked to obesity-related insulin resistance, which initially is compensated by enhanced capacity of β cells to secrete insulin. However, in a large subset of obese and insulin-resistant individuals, these compensatory mechanisms are impaired, leading to reduced β cell mass and function and culminating in manifest diabetes (Kahn et al., 2006). β cell damage and insulin resistance appear to be at least partially triggered by inflammatory, oxidative, and endoplasmic reticulum stress-induced pathways including the mitogen-activated protein kinase (MAPK) signaling cascade (Wellen and Hotamisligil, 2005). Indeed, activation of the MAPK c-*jun* N-terminal kinase (JNK) represents a central signal transduction event promoting peripheral insulin resistance, suppressing insulin production and secretion, and increasing apoptosis of islet cells (Hirosumi et al., 2002; Kaneto et al., 2004).

The role of the p38 MAPKs (which are closely related to JNK) in these processes remains poorly understood. p38 activity has been reported to be increased in insulin-resistant peripheral tissues from diabetic patients (Koistinen et al., 2003). Moreover, in vitro data have demonstrated that p38 activation upon exposure to TNF-α, free fatty acids, and oxidative stress impairs insulin signaling in adipocytes and skeletal muscle cells through mechanisms very similar to those described for JNK (de Alvaro et al., 2004). Finally, activation of p38 appears to trigger pancreatic β cell dysfunction and apoptosis in response to oxidative stress and cytokines in vitro (Makeeva et al., 2006). Verification of an involvement of p38 in metabolic diseases in vivo is complicated by the existence of four different *p38* genes, *p38α*, *p38β*, *p38γ*, and *p38δ*.

To date, the best-characterized p38 isoform is p38α. It has been shown that *p38α* knockout mice die at midgestation, likely because of a defective placental organogenesis (Adams et al., 2000; Mudgett et al., 2000; Tamura et al., 2000). Recently, it has been demonstrated that p38α is a fundamental regulator of cellular proliferation and carcinogenesis in vivo (Hui et al., 2007; Ventura et al., 2007). p38α is also required to mediate an inflammatory response in macrophages (Kang et al., 2008). On the basis of inhibitor studies, it was postulated that p38α and p38β functionally cooperate in the context of inflammatory processes. However, *p38β*-specific knockout mice revealed no differences in several in vivo and in vitro models of inflammation (Beardmore et al., 2005).

The least-characterized isoforms are p38γ and p38δ. p38γ is predominantly expressed in skeletal muscle and heart, and *p38γ*-deficient myoblasts exhibited an attenuated cell-to-cell fusion capacity in vitro (Perdiguero et al., 2007). The isoform p38δ shares approximately 60% homology with the other p38 family members and about 40% homology with other MAPKs. Like p38α, p38δ is activated by various stress stimuli, including inflammatory cytokines and oxidative stress (Jiang et al., 1997). Recent in vitro studies have demonstrated that p38δ might be involved in keratinocyte differentiation and PKCδ-dependent keratinocyte apoptosis (Efimova et al., 2004), as well as the progression of neurodegenerative disorders referred to as tauopathies (Feijoo et al., 2005). However, no specific in vivo functions of p38δ have been reported thus far.

To address such functions, we generated *p38δ* null mice. Remarkably, these mice displayed improved glucose tolerance due to enhanced insulin exocytosis from pancreatic β cells. Moreover, inactivation of *p38δ* protected against hyperlipidemia-induced insulin resistance and oxidative stress-imposed β cell apoptosis. At the molecular level, we discovered that p38δ exerts an inhibitory phosphorylation on protein kinase D 1 (PKD1), a kinase that regulates both stimulated insulin secretion and pancreatic β cell survival. We propose that p38δ represents a critical regulator of glucose homeostasis in vivo.

## Results

### Enhanced Glucose Tolerance in *p38δ* Null Mice Due to Increased Insulin Exocytosis from Pancreatic β Cells

**Figure 1:**
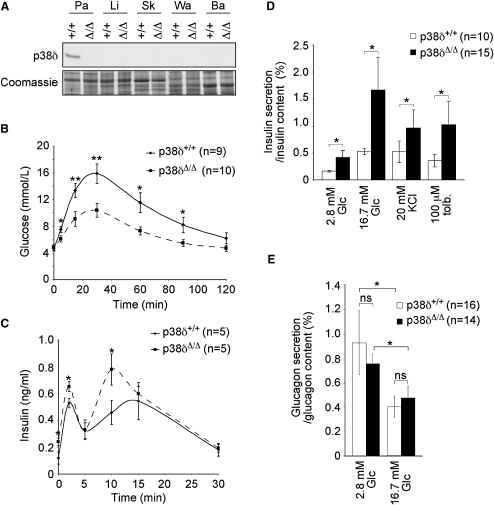
Mice Lacking *p38δ* Show Improved Glucose Tolerance and Enhanced Insulin Secretion (A) Western blotting revealed expression of p38δ in wild-type (+/+) pancreas but not in liver (Li), skeletal muscle (Sk), and white (Wa) and brown (Ba) adipose tissue compared to tissues from *p38δ* null (Δ/Δ) mice. Coomassie blue staining was used to confirm equal loading. (B) Glucose tolerance test (GTT) in mice with the indicated genotypes (^∗^p < 0.05, ^∗∗^p < 0.01). (C) Parallel measurements of serum insulin during the GTT in mice with the indicated genotypes (^∗^p < 0.05). (D) Insulin release in response to indicated stimuli (Glc, glucose; KCl, potassium chloride; tolb., tolbutamide) in isolated islets with the indicated genotypes (^∗^p < 0.05). (E) Glucagon secretion in response to 2.8 mM and 16.7 mM glucose in islets isolated with the indicated genotypes (ns, not significant). All error bars indicate ± SEM.

To address in vivo functions of p38δ in metabolism, we generated *p38δ* floxed mice and crossed them with *protamine* promoter-driven Cre recombinase-expressing mice, a male germline deleter strain (O'Gorman et al., 1997). With this approach, we obtained *p38δ* null mice (*p38δ*^Δ/Δ^ mice) and corresponding wild-type control littermates (*p38δ*^+/+^ mice) (Figure S1 available online). *p38δ*^Δ/Δ^ mice presented with normal general health, viability, fecundity, body composition, and body weight (data not shown). Assessment of the expression of p38δ in organs involved in glucose homeostasis revealed that p38δ was abundantly expressed both at the mRNA and protein level and in similar amounts in the exo- and endocrine pancreas. In contrast, no expression of p38δ was observed in insulin-sensing organs such as adipose tissue and liver. Very low amounts of *p38δ* mRNA were detected in skeletal muscle (Figure 1A and Figure S2A).

The observed expression pattern prompted us to investigate the role of p38δ in glucose homeostasis. *p38δ*^Δ/Δ^ mice fasted for 16 hr showed a significantly enhanced glucose tolerance compared to *p38δ*^+/+^ mice (Figure 1B), while insulin sensitivity was equal in *p38δ*^Δ/Δ^ and *p38δ*^+/+^ mice (Figure S2B). Although lower glucose levels were attained in *p38δ*^Δ/Δ^ mice after the glucose challenge, circulating insulin levels were increased compared to *p38δ*^+/+^ control mice. The glucose challenge elicited a biphasic insulin response; both initial first phase and subsequent second phase were enhanced in *p38δ*^Δ/Δ^ mice (Figure 1C).

Enhanced insulin release in *p38δ^Δ/Δ^* mice was not the consequence of alterations in the β cell mass, in the pancreatic islet architecture, or in insulin content (Figure S3). Transmission electron microscopy further revealed that the volume density and distribution of both pale (immature) and dense core (mature) secretory granules were similar in β cells in islets of *p38δ^+/+^* and *p38δ^Δ/Δ^* mice (Table S1).

To test whether enhanced insulin secretion in *p38δ^Δ/Δ^* mice primarily reflects an intrinsic islet/β cell effect that is independent of the action of incretins or innervation, we isolated pancreatic islets from *p38δ^Δ/Δ^* and *p38δ*^+/+^ mice and tested their capacity to secrete insulin in vitro. Knockout islets released more insulin both under basal (2.8 mM glucose) and glucose-stimulated (16.7 mM glucose) conditions compared to control islets (Figure 1D). By contrast, glucagon secretion was unaffected in *p38δ^Δ/Δ^* islets, and high glucose (16.7 mM) inhibited the release of the hormone to the same extent in both wild-type and knockout islets (Figure 1E). These data suggest that lack of *p38δ* improves glucose tolerance and enhances insulin secretion by a direct and β cell-specific mechanism.

### Enhanced Insulin Exocytosis in *p38δ*-Deficient Pancreatic β Cells Is Independent of Differences in Glucose Metabolism, K_ATP_ Closure, and Ca^2+^ Influx

We next addressed at which step p38δ interferes with the insulin secretory pathway in pancreatic β cells. In *p38δ*^Δ/Δ^ islets, insulin secretion elicited by either 20 mM KCl (to evoke depolarization of membrane) or 100 μM tolbutamide (to close ATP-regulated K^+^ channels and elicit electrical activity) was enhanced compared to control islets (Figure 1D).

To determine whether ablation of *p38δ* affects Ca^2+^ influx, we measured intracellular calcium concentrations ([Ca^2+^]_i_) in response to glucose and KCl in islets isolated from *p38δ*^Δ/Δ^ and *p38δ*^+/+^ mice. The average [Ca^2+^]_i_ was similar in the two groups of islets both under basal conditions (2.8 mM glucose) and after stimulation by either glucose (16.7 mM) or KCl (20 mM in the presence of 2.8 mM glucose) (Figures S4A and S4B). Likewise, whole-cell Ca^2+^ currents were comparable in single β cells of *p38δ*^Δ/Δ^ and *p38δ*^+/+^ littermates (Figures S4C and S4D). The fact that insulin secretion was enhanced in *p38δ*^Δ/Δ^ islets despite similar [Ca^2+^]_i_ levels suggests that p38δ acts directly at the level of exocytosis.

**Figure 2:**
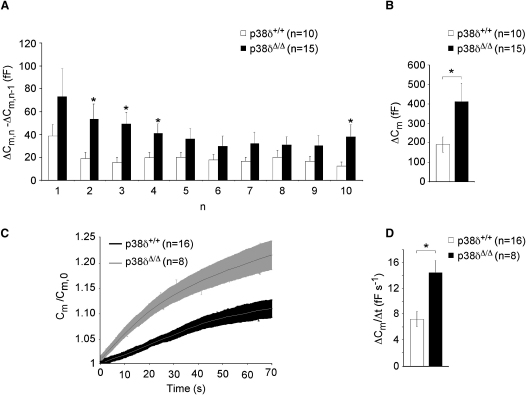
Enhancement of Exocytosis in *p38δ*-Deficient Pancreatic β Cells Occurs Downstream of Calcium Influx (A) A train of ten successive 500 ms depolarizations from −70 to 0 mV increased capacitance (in femtofarad [fF]) in pancreatic β cells with the indicated genotypes (^∗^p < 0.05). (B) Average cumulative increase of capacitance (^∗^p < 0.05). (C) Changes in cell capacitance (C_m_) when exocytosis was elicited by intracellular application of 1.5 μM free [Ca^2+^]_i_ via the recording electrode capacitance in pancreatic β cells with the indicated genotypes. Values have been normalized to the resting cell capacitance (C_m,0_), which was 4.6 ± 0.4 pF (n = 8) and 4.6 ± 0.2 pF (n = 16) in *p38δ*^Δ/Δ^ and *p38δ*^+/+^ cells, respectively. Data are presented as the mean values (central lines) and ± SEM (shaded areas). (D) Steady-state average rate of capacitance change (in fF/s) for cells measured over a 60 s period (^∗^p < 0.05). All error bars indicate ± SEM.

To address whether exocytosis is enhanced in *p38δ*-deficient β cells, we performed high-resolution capacitance measurements of exocytosis on single β cells (Gopel et al., 2004). A train of ten depolarization steps from −70 mV to 0 mV evoked larger responses in *p38δ^Δ/Δ^* than in control β cells, resulting in a 2-fold larger increase of membrane capacitance in *p38δ^Δ/Δ^* cells (Figures 2A and 2B). Exocytosis was enhanced approximately equally in *p38δΔ/Δ* compared to *p38δ*+/+ β cells throughout the trains of depolarization. The rate of capacitance increase in cells lacking *p38δ* was also higher than that of wild-type cells when exocytosis was elicited by clamping [Ca^2+^] at 1.5 μM, a condition that bypasses any effects on Ca^2+^ entry (Figures 2C and 2D). Collectively, these results indicate that the enhanced insulin secretion from *p38δ*-deficient islets is not caused by differences in glucose metabolism, K_ATP_ closure, or [Ca^2+^]_i_ homeostasis between knockout and control β cells. Rather, ablation of p38δ influences insulin secretion by a direct effect on the exocytotic machinery that is exerted downstream of [Ca^2+^]_i_ elevation.

### p38δ Exerts an Inhibitory Phosphorylation on Protein Kinase D 1

**Figure 3:**
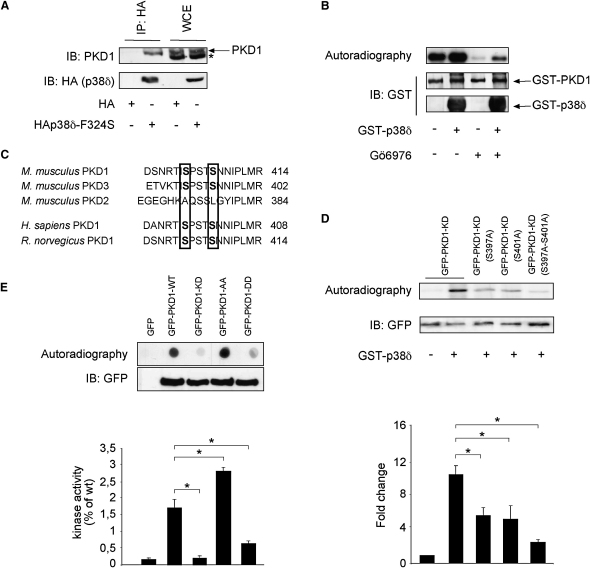
p38δ Interacts with PKD1 and Phosphorylates It at Ser 397 and 401 (A) Western blot with indicated immunoprecipitates (IP) and whole-cell extracts (WCE) from INS1 cells stably transfected with indicated constructs confirms physical interaction with endogenous PKD1 (^∗^unspecific band). (B) In vitro kinase assay with and without recombinant p38δ and recombinant *E. coli*-derived GST-tagged PKD1 in the presence and absence of the PKC inhibitor Gö6976. (C) Sequence alignment of the region around S403-407 of mouse PKD1, PKD3, and PKD2, as well as human and rat PKD1. Conserved residues (S403-407 in mPKD1, S397-401 in hPKD1) are indicated by rectangles. (D) In vitro kinase assay with recombinant p38δ and indicated immunoprecipitated proteins. Quantification of autoradiography with corresponding bars positioned under bands (^∗^p < 0.05). (E) In vitro kinase assay with indicated immunoprecipitated proteins using CREBtide as a substrate. CREBtide was spotted, while immunopreciptates were tested for equal loading by western blotting. Quantification of radiography using a scintillation counter with corresponding bars positioned under spots (^∗^p < 0.05). All error bars indicate ± SEM.

We next investigated a possible molecular mechanism by which p38δ attenuates insulin exocytosis using an unbiased proteomic approach. We ectopically expressed a hemagglutinin (HA)-tagged constitutively active form of p38δ, obtained by substitution of phenylalanine 324 with serine (F324S) as previously described (Askari et al., 2007), in 293T cells. Constitutive activity was tested in an in vitro kinase assay using recombinant HIS-tagged ATF-2 as a substrate (Figure S5A). HA immunoprecipitates from HA-p38δ^F324S^ and HA-expressing cells were analyzed by liquid chromatography-tandem mass spectrometry (LC-MS/MS). A number of putative interactors were obtained among which protein kinase D 1 (PKD1) was most frequently found (represented by a total of 36 unique peptides) (Table S2). PKD is necessary for biogenesis of trans-Golgi network (TGN) to cell surface transport carriers (Bard and Malhotra, 2006) and has been shown to be a positive regulator of secretion in neuroendocrine cells (Li et al., 2004). The interaction between p38δ and PKD1 was confirmed by coimmunoprecipitation experiments in 293T cells (Figure S5B). HA pulldowns also contained endogenous PKD1 in INS1 cells (rat insulinoma-derived pancreatic β cell line) stably expressing HA-p38δ^F324S^ but not in cells expressing HA alone (Figure 3A).

We then tested the ability of p38δ to directly phosphorylate PKD1 in vitro. Active recombinant p38δ phosphorylated immunoprecipitated human GFP-tagged wild-type PKD1 (GFP-PKD1-WT) and kinase-dead PKD1 (GFP-PKD1-KD) (Figure S5C), as well as Sf9 cell-derived (Figure S5D) and *E. coli*-expressed recombinant human GST-PKD1 (Figure 3B). Bacterial PKD1 showed high basal activity that caused high autophosphorylation. However, enhancement of phosphorylation by addition of p38δ became more evident when the PKD inhibitor Gö6976 was added to the reactions to reduce autophosphorylation. The LC-MS/MS analysis of in vitro phosphorylated immunoprecipitated murine GST-PKD1 identified an autophosphorylation and a 14-3-3 binding site (Ser^203^ and Ser^206^) (Zhang et al., 2005), one of the activating dual phosphorylation sites (Ser^748^), and a protein kinase C-dependent transphosporylation site (Ser^255^) (Vertommen et al., 2000) (Figure S6). p38δ specifically phosphorylated Ser^403^, a p38 MAPK consensus site (SP/TP), as well as the closely located Ser^407^ residue (Figure S7). LC-MS/MS analysis of the HA-p38δ^F324S^ immunoprecipitates from 293T cells confirmed Ser^403^ phosphorylation on endogenous PKD (data not shown). Both phosphorylation sites are conserved between mouse (Ser^403^ and Ser^407^), rat, and human (Ser^397^ and Ser^401^) and can be found in PKD1 and PKD3 but not in PKD2 (Figure 3C). PKD1 is the predominant isoform expressed in pancreatic β cells (data not shown).

We next generated the corresponding single and double serine to alanine mutants of kinase-dead PKD1 and double serine to alanine mutants of wild-type PKD1 (GFP-PKD1-AA), as well as double serine to aspartate mutants of wild-type PKD1 (GFP-PKD1-DD). Mutations of either Ser^397^ or Ser^401^ to alanines significantly reduced p38δ-dependent phosphorylation in vitro, and mutation of both sites diminished autoradiography almost to background levels (Figure 3D). The ability of kinases to phosphorylate the substrate CREBtide was assayed in vitro. Autoradiography of spotted CREBtide from reactions with GFP-PKD1-KD was markedly reduced compared to reactions with GFP-PKD1-WT, while no signal could be detected with CREBtide from reactions with GFP only. Importantly, CREBtide autoradiography from reactions containing GFP-PKD1-AA was enhanced, whereas it was markedly reduced in reactions with GFP-PKD1-DD compared to reactions with GFP-PKD1-WT. Quantification of radiography of purified CREBtide with a scintillation counter confirmed respective kinase activities (Figure 3E). These results suggest that PKD1 is a direct substrate of p38δ and that p38δ-dependent phosphorylation of PKD1 constitutes an inhibitory modification.

### Enhanced Activity of PKD and Altered Golgi Organization in Pancreatic β Cells Lacking p38δ

**Figure 4:**
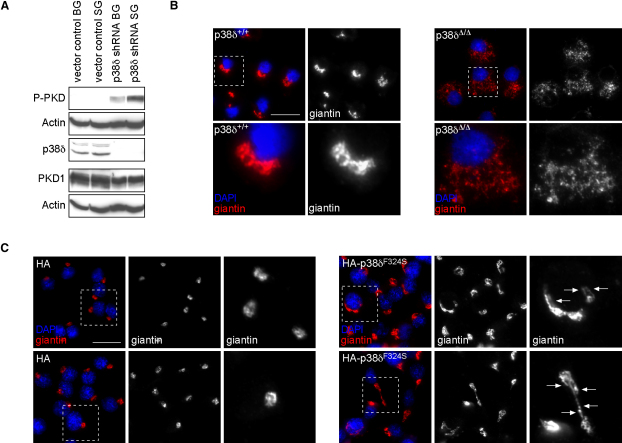
Increased Activity of PKD Leads to Altered Golgi Organization in *p38δ*-Deficient β Cells (A) Activity of protein kinase D (PKD) was determined by western blotting with an antibody against the activatory phosphorylation sites (serines 744 and 748) (BG, basal glucose and SG, stimulatory glucose levels). Immunoblotting with an antibody against Actin was used to determine equal loading of phopho-PKD and total PKD1 blots. (B) Immunofluorescence with antibodies against giantin (red) in pancreatic β cells with the indicated genotypes. (C) Immunofluorescence with antibodies against giantin (red) in INS1 cells stably expressing HA or HA-p38δ^F324S^ cultured in the presence of stimulatory glucose. Expression of p38δ^F324S^ led to formation of tubular protrusions from the Golgi complex (arrows). (B and C) Dashed boxes outline the areas that were magnified. Nuclear DNA was stained with DAPI (blue). Scale bars represent 10 μm.

We proceeded to test whether lack of *p38δ* results in increased activity of PKD in pancreas by assessing PKD autophosphorylation (serine 916). The activity was markedly enhanced in *p38δ*^Δ/Δ^ compared to *p38δ*^+/+^ pancreas (Figure S8). To confirm increased PKD activity in β cells, we generated MIN6 cells (murine insulinoma-derived pancreatic β cell line) stably expressing small hairpins (shRNA) against *p38δ*. Consistently, lack of *p38δ* enhanced insulin secretion also in MIN6 cells (see below). Indeed, a marked increase in activatory PKD phosphorylation was also observed in MIN6 cells lacking *p38δ* compared to control cells, which was further increased in *p38δ*-deficient cells stimulated by glucose (Figure 4A).

Activation of PKD induces membrane fission at the TGN, which can be monitored by altered localization of Golgi marker proteins by immunofluorescence (Bossard et al., 2007; Liljedahl et al., 2001). Indeed, the Golgi markers giantin, furin convertase, and GM130 exhibited a diffuse distribution in *p38δ*^Δ/Δ^ primary pancreatic β cells, whereas a characteristic crescent-shaped staining was found in *p38δ*^+/+^ cells (Figure 4B and Figure S9). Altered Golgi organization was confirmed in MIN6 cells lacking *p38δ* by immunofluorescence stainings of giantin (Figure S10). Conversely, INS1 cells ectopically expressing HA-p38δ^F324S^ showed tubular protrusions from the Golgi apparatus reminiscent of inhibited TGN membrane fission (Figure 4C). The latter cellular phenotype correlated with suppressed stimulated insulin secretion in INS1 cells (see below). Collectively, these data indicate that lack of *p38δ* leads to constitutive PKD activity and enhanced membrane fission at the TGN in pancreatic β cells, whereas increased p38δ activity has the opposite effects.

### Inhibitors of Phospolipase C and Conventional PKCs Restore Insulin Secretion in *p38δ*-Deficient Islets

**Figure 5:**
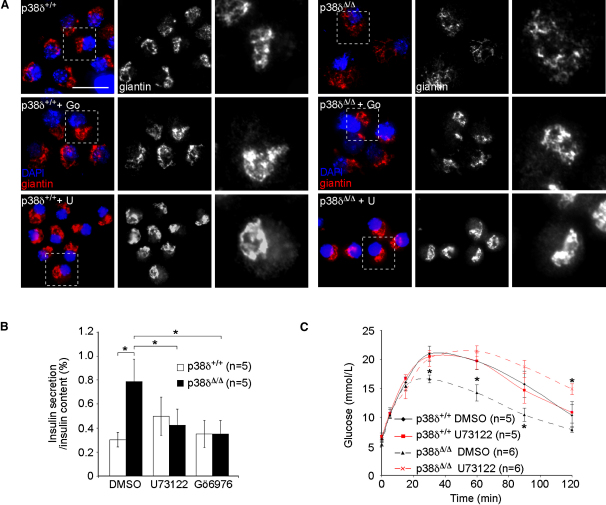
Restoration of Insulin Secretion in *p38δ*^Δ/Δ^ Islets and Glucose Tolerance in *p38δ*^Δ/Δ^ Mice by Pharmacological Inhibition of PKD (A) Immunofluorescence using antibodies against giantin (red) in pancreatic β cells with the indicated genotypes exposed to Gö6976 (Go) or U73122 (U) as indicated. Nuclear DNA was stained with DAPI (blue). Dashed boxes outline the areas that have been magnified. The scale bar represents 10 μm. (B) Glucose (16.7 mM)-stimulated insulin secretion from islets with the indicated genotypes exposed to DMSO, U73122, and Gö6976 (^∗^p < 0.05). (C) Glucose tolerance test (GTT) in mice with the indicated genotypes treated with DMSO or U73122 as indicated (^∗^p < 0.05). All error bars indicate ± SEM.

Generation of diacylglycerol (DAG) by phosphatidyl-inositol-specific phospholipases C (PI-PLCs; PLC, phospolipase C) activates PKD, which is subsequently recruited to the TGN, where it promotes membrane fission (Diaz Anel, 2007). Pharmacological inhibition of this pathway is expected to reverse cellular phenotypes and related effects caused by deletion of *p38δ*. To test this, we used the PI-PLC inhibitor U73122 and Gö6976, a potent inhibitor of PKD and conventional protein kinases C (PKCs) (Haxhinasto and Bishop, 2003). Both U73122 and Gö6976 resulted in relocalization of giantin to the TGN in *p38δ*^Δ/Δ^ pancreatic β cells, whereas none of the compounds altered normal localization of giantin in *p38δ*^+/+^ cells (Figure 5A). These cellular effects echoed those on glucose-evoked (16.7 mM) insulin secretion: none of the compounds had any effect on insulin secretion from *p38δ*^+/+^ islets, but the enhancement seen in *p38δ*^Δ/Δ^ islets was abolished (Figure 5B). Importantly, peritoneal injections of U73122 decreased glucose tolerance of *p38δ*^Δ/Δ^ mice to that observed in dimethyl sulfoxide (DMSO)-treated *p38δ*^+/+^ controls, without modifying glucose tolerance of the control animals (Figure 5C). Thus, inhibition of PKD reverses the effects of *p38δ* ablation on Golgi organization, insulin secretion, and glucose tolerance.

### p38δ Negatively Regulates PKD-Mediated Stimulated Insulin Secretion

**Figure 6:**
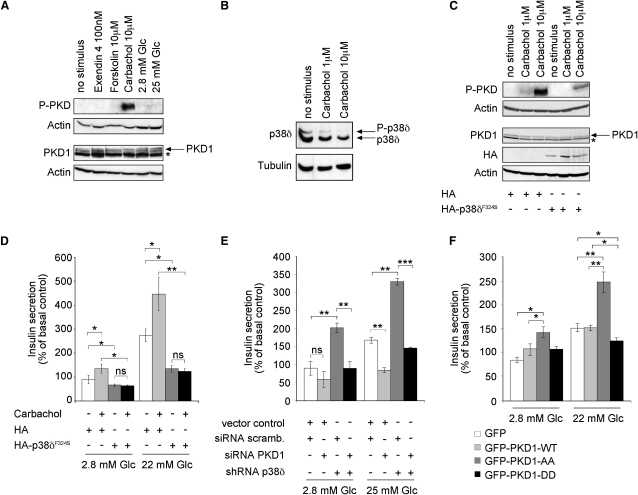
p38δ Suppresses PKD-Mediated Stimulated Insulin Secretion (A) Western blot to determine the activity of protein kinase D (PKD) in INS1 cells in response to indicated stimuli using an antibody against the activatory phosphorylation sites (serines 744 and 748) (^∗^unspecific band). (B) SDS-PAGE with polyacrylamide-bound Mn^2+^-Phos-tag to measure the activity of p38δ in nonstarved INS-1 cells in response to carbachol. Phosphorylated p38δ (P-p38δ) migrated slower than unmodified p38δ. (C) Western blot to determine the activity of protein kinase D (PKD) in INS1 cells stably transfected with indicated constructs in response to carbachol (^∗^unspecific band). (D) Insulin secretion from INS1 cells stably expressing HA or HA-p38δ^F324S^ in response to carbachol under basal and stimulatory glucose levels (^∗^p < 0.05 and ^∗∗^p < 0.01; ns, not significant). (E) Insulin secretion in response to basal and stimulatory glucose from MIN6 cells stably expressing shRNA against p38δ or a control vector with simultaneous siRNA-mediated knockdown of PKD1 or transfection of a scrambled siRNA (^∗^p < 0.05, ^∗∗^p < 0.01, and ^∗∗∗^p < 0.001; ns, not significant). (F) Insulin secretion in response to basal and stimulatory glucose from INS1 cells stably expressing GFP, GFP-tagged WT, and mutated forms of PKD (^∗^p < 0.05 and ^∗∗^p < 0.01). (D–F) All experiments were performed three times independently. All error bars indicate ± SEM.

PKD resides in the G_q_ protein-coupled receptor (G_q_PCR) pathway, which in β cells is known to be strongly activated by the insulin secretagogue acetylcholine (Gilon and Henquin, 2001). Indeed, activity of PKD was markedly increased upon stimulation with the acetylcholine analog carbachol. By contrast, exendin-4 (a GLP-1 analog) (Drucker, 2006), forskolin (Wiedenkeller and Sharp, 1983), and glucose did not detectably activate PKD (Figure 6A). The activity of p38δ in response to carbachol was examined in INS1 cells with the Phos-tag technology (Kinoshita et al., 2006). Both phosphorylated p38δ and nonphosphorylated p38δ declined in INS1 cells (Figure 6B), indicating that carbachol-induced activation of PKD1 correlates with p38δ inhibition.

We next assessed the role of PKD1 in insulin secretion. PKD1 was specifically inactivated in INS1 cells by siRNA-mediated knockdown with two independent oligonucleotide sequences as compared to a scrambled control siRNA (Figure S11A). Although an induction of PKD activity was only apparent upon stimulation with carbachol, deletion of PKD1 in INS1 cells completely blocked insulin secretion in response to both carbachol and glucose (Figure S11B), indicating a general requirement of PKD1 in stimulated insulin secretion. We went on to investigate whether PKD activity and insulin secretion in response to glucose and carbachol is p38δ dependent. Carbachol-stimulated activation of PKD was markedly reduced in INS1 cells expressing HA-p38δ^F324S^ compared to cells expressing HA alone (Figure 6C). Importantly, expression of HA-p38δ^F324S^ in INS1 cells completely blocked insulin secretion in response to carbachol and also attenuated secretion in response to glucose (Figure 6D).

We subsequently tested whether inactivation of PKD1 reverses insulin release in the absence of *p38δ* to levels seen in wild-type cells. For this purpose, we used MIN6 cells stably expressing shRNA against *p38δ* as well as control cells expressing an empty vector and simultaneously performed siRNA against PKD1. Efficient knockdown of p38δ and PKD1 was confirmed by Western blotting (Figure 4A and Figure S12A). Compared to control cells, MIN6 cells lacking *p38δ* showed enhanced insulin secretion in the presence of basal (2.8 mM) and of stimulatory glucose levels (25 mM), whereas knockdown of *Pkd1*, as seen in INS1 cells (Figure S11), led to blockage of glucose-stimulated insulin secretion. Inactivation of *Pkd1* in MIN6 cells lacking *p38δ* lowered insulin secretion to levels observed in control MIN6 cells (Figure 6E).

Finally, to assess the functional relevance of p38δ-dependent PKD1 phosphorylation, we generated INS1 cells ectopically expressing mutated forms of PKD1 (Figure S12B). Importantly, glucose-induced insulin secretion was suppressed by expression of GFP-PKD1-DD under stimulatory glucose conditions, whereas it was markedly increased by expression of GFP-PKD1-AA under both basal and stimulatory glucose conditions compared to GFP- and GFP-PKD1-WT-expressing cells (Figure 6F). Altogether, these data suggest that p38δ suppresses PKD-mediated stimulated insulin secretion.

### *p38δ* Deficiency Protects against Insulin Resistance and Pancreatic β Cell Failure

**Figure 7:**
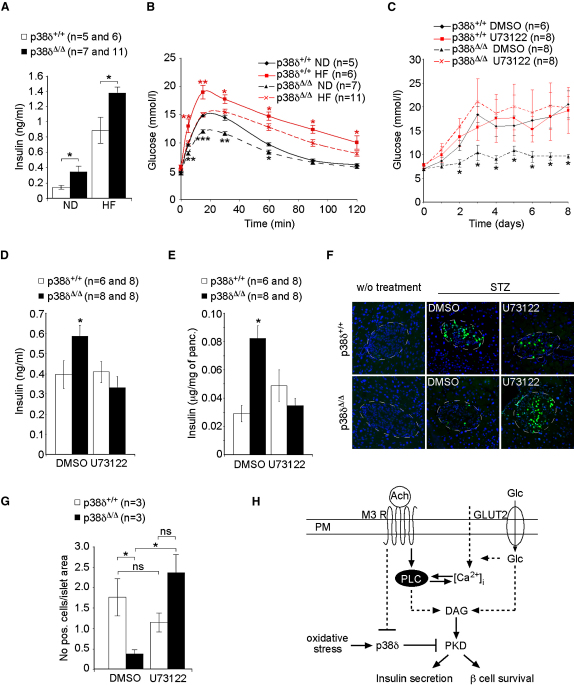
*p38δ* Deficiency Protects Mice from Diabetes (A) Blood insulin levels in fasted mice with the indicated genotypes fed a high-fat diet (HF) or a normal diet (ND) after 12 weeks (^∗^p < 0.05). (B) Glucose tolerance test (GTT) in mice with the indicated genotypes fed a high-fat diet (HF) or a normal diet (ND) (^∗^p < 0.05, ^∗∗^p < 0.01, and ^∗∗∗^p < 0.001). (C) Blood glucose levels in mice with the indicated genotypes treated with STZ and DMSO or U73122 (^∗^p < 0.05). (D) Blood insulin levels and (E) total pancreatic insulin content in mice with the indicated genotypes treated with DMSO or U73122 8 days after STZ injections. (F) TUNEL stain of islets of mice with the indicated genotypes treated with STZ and DMSO or U73122 and of mice without treatment (w/o treatment). (G) Quantification of TUNEL-positive cells in relation to islet area (^∗^p < 0.05; ns, not significant). (H) Model: Acetylcholine (Ach)- and glucose (Glc)- induced pathways leading to increased PKD activity are indicated (M3 R, muscarinic receptor subtype M3; GLUT2, glucose transporter 2; DAG, Diacylglycerol; PLC, phospholipase C). PKD activation leads to insulin secretion and promotes β cell survival. In diabetes, oxidative stress-induced activation of p38δ might interfere with PKD-mediated signaling, leading to impaired pancreatic β cell function. All error bars indicate ± SEM.

To challenge the insulin secretion capacity of islets, we placed *p38δ*^Δ/Δ^ and *p38δ*^+/+^ mice on a high-fat diet, a widely used model for insulin resistance (Biddinger and Kahn, 2006). Although insulin sensitivity was reduced by this protocol in both genotypes, it remained significantly better in *p38δ*^Δ/Δ^ compared to *p38δ*^+/+^ mice (Figure S13A). Improved insulin sensitivity in *p38δ*^Δ/Δ^ mice was associated with a moderately reduced body weight gain (Figure S13B). As expected, insulin resistance led to a marked hyperinsulinemia in both *p38δ*^Δ/Δ^ and *p38δ*^+/+^ mice compared to mice on a normal diet. However, *p38δ*^Δ/Δ^ mice showed significantly enhanced fasting insulin levels on a high-fat diet compared to *p38δ*^+/+^ mice, indicating that they maintain their enhanced capacity to secrete insulin also under insulin-resistant conditions (Figure 7A). No significant differences in islet growth in *p38*δ*^+/+^* and *p38*δ^Δ/Δ^ mice in response to a high-fat diet could be observed (Figure S14).

Overall, differences in insulin sensitivity as well as insulin levels resulted in a significantly improved glucose tolerance in *p38δ*^Δ/Δ^ mice on a high-fat diet (Figure 7B). Thus, lack of *p38δ* provides protection against lipid-induced glucose intolerance.

Oxidative stress is known to contribute to pancreatic β cell loss in insulin resistance-related diabetes mellitus (Fridlyand and Philipson, 2006). Streptozotocin (STZ)-induced oxidative stress is a widely used model to trigger pancreatic β cell failure in vivo (Le May et al., 2006). We confirmed that STZ activates p38δ in pancreatic β cells (Figure S15A). As expected, glucose levels increased in *p38δ^+/+^* mice after STZ injection, reaching up to 15 mmol/l concentrations at day 8. However, no such increase was observed in *p38δ^Δ/Δ^* mice, in which plasma glucose levels remained stable and around 7 mmol/l throughout the observation period (Figure S15B). Plasma insulin levels and pancreatic insulin content were significantly higher in *p38δ*^Δ/Δ^ mice than in *p38δ*^+/+^ mice treated with STZ (Figures S15C and S15D). The involvement of PKD in the protection of β cells in *p38δ*^Δ/Δ^ mice was tested with the inhibitor U73122. In *p38δ*^Δ/Δ^ mice, U73122 increased plasma glucose and lowered insulin to the same levels as in *p38δ*^+/+^ mice after STZ injections (Figures 7C–7E). The inhibitor had no additive effect in *p38δ*^+/+^ mice.

TUNEL staining revealed that whereas STZ-induced hyperglycemia in *p38δ*^+/+^ mice was associated with a high rate of β cell apoptosis, the rate of apoptosis was 5-fold lower in *p38δ*^Δ/Δ^ mice. The protective effect of lack of *p38δ* on apoptosis was abolished by U73122 (Figures 7F and 7G). These data raise the interesting possibility that p38δ-imposed inhibition of PKD might contribute to β cell dysfunction and destruction in diabetic subjects.

## Discussion

A nonredundant and specific in vivo function of the δ isoform of the MAPK family p38 has not been elucidated so far. We now provide compelling evidence that p38δ represents a key regulator of pancreatic β cell function. Our work supports a negative regulatory role for p38δ in stimulated insulin secretion through inhibition of PKD1 and regulation of exocytosis. Moreover, immoderate suppression of PKD activity by p38δ may also contribute to β cell dysfunction in diabetic subjects.

### The p38δ-PKD1 Pathway Regulates Stimulated Insulin Secretion from Pancreatic β Cells

We demonstrate that ablation of *p38δ* activates PKD1, and thereby enhances insulin secretion and consequently improves glucose tolerance. Furthermore, we show that the acetylcholine analog carbachol strongly activates PKD in pancreatic β cells. This physiological function of PKD1 can be completely blocked by enhancement of p38δ activity. Acetylcholine represents the major neurotransmitter of the peripheral parasympathetic nervous system, and its binding on muscarinic acetylcholine receptors located on the pancreatic β cells potentiates secretion of insulin (Gautam et al., 2006). Indeed, muscarinic receptors belong to G_q_ protein-coupled receptors stimulating PLC to produce inositol 1,4,5-trisphosphate (IP_3_) and DAG, the latter of which activates numerous PKC family members, including PKD (Oancea et al., 2003) (Figure 7H).

Importantly, PKD1 deletion also blocks insulin secretion in response to glucose. PKD activity upon glucose stimulation could not be detected in wild-type β cells. However, in β cells lacking *p38δ*, in which PKD activity is constitutively enhanced, a 2-fold increase of PKD activity was seen upon glucose stimulation (Figure 4A). It has been reported that glucose generates DAG in β cells (Peter-Riesch et al., 1988). This can either occur by a direct effect of glucose metabolism or be secondary to glucose-induced increases in [Ca^2+^]_i_- and Ca^2+^-induced activation of PLC (Thore et al., 2007) (Figure 7H). It is therefore tempting to speculate that activity of PKD is also rising in response to glucose in wild-type cells but is probably below our detection limit. Altogether, our data support a key role of the p38δ-PKD1 pathway in stimulated insulin secretion.

### The p38δ-PKD1 Pathway Regulates Proximal and Distal Steps of Exocytosis in Pancreatic β Cells

The findings in capacitance experiments conform to functions of the herein identified p38δ target, PKD1. One of the most established roles of PKD is to promote fission of cell surface-destined transport carriers from the TGN (Bossard et al., 2007; Liljedahl et al., 2001). Enhanced membrane fission at the TGN in the absence of *p38δ* most likely accounts for late effects (pulses 5 to 10) seen in capacitance experiments.

Importantly, it has been demonstrated that ectopic expression of constitutively active PKD is sufficient to promote secretion of neurotensin, implying that PKD in addition to its function at the TGN primes vesicles for efficient transport and immediate fusion (Li et al., 2004). Accordingly, enhanced PKD activity might also explain early effects (pulses 1 to 4) observed in capacitance experiments. Indeed, a very recent report suggests that PKD-mediated secretion of neurotensin requires its target, Kidins220, the latter of which is proposed to regulate more distal steps of exocytosis (Li et al., 2008). Moreover, although not specifically reported for PKD so far, PKCs downstream of DAG were shown to increase the efficiency of Ca^2+^ on insulin exocytosis independent of a rise in cytosolic free Ca^2+^ levels, a mechanism that also underlies acetylcholine-mediated insulin secretion (Gilon and Henquin, 2001). The observed increase of exocytosis is also reminiscent of that previously reported for the PKC/PKD activator PMA, the latter effect also being exerted distally to the elevation of [Ca^2+^]_i_ (Ammala et al., 1994).

Overall, our data indicate that stimulated insulin secretion is increased, at least partially, through enhancement of the efficiency of TGN function, a cellular mechanism, which has not been reported so far in this context.

### The p38δ-PKD1 Axis Might be Pivotal in Maintaining β Cell Function in Diabetic Conditions

In our study, we have challenged the secretory capacity of pancreatic islets in knockout mice with a high-fat diet, which induces peripheral insulin resistance, leading to adaptive hyperinsulinemia. Interestingly, *p38δ* null mice developed less severe insulin resistance. Strikingly, *p38δ* null mice on a high-fat diet became hyperinsulinemic, reaching fasting insulin levels that were significantly higher than those in high fat-fed control mice. The relative importance of increased insulin sensitivity and enhanced insulin secretion to the overall improved glucose tolerance needs to be further investigated in the future.

Additionally, we have shown that p38δ plays yet another key function in the β cell: the regulation of β cell destruction during oxidative stress, which is a key pathogenic mechnism in both, type 1 and type 2 diabetes mellitus (Muoio and Newgard, 2008). Strikingly, *p38δ* knockout mice were protected against STZ-imposed oxidative stress in β cells. Importantly, this phenotype also appears to be dependent on PKD activity. This is in agreement with the report demonstrating that PKD activation is protective against oxidative stress-induced apoptosis through activation of NF-κB (Storz and Toker, 2003). Even though p38δ fine-tunes PKD-mediated insulin secretion in normal physiologic settings, in pathological situations of gradually increasing cellular oxidative stress, p38δ activity might exceed and PKD activity may drop to levels affecting insulin secretion as well as β cell survival (Figure 7H).

There is accumulating evidence that forcing β cells to secrete insulin by currently used drugs, including sulfonylureas, ultimately results in pancreatic β cell failure (Aston-Mourney et al., 2008). An ideal pharmacological diabetes therapy should therefore combine an insulinotropic effect with protection against β cell failure. Our data suggest that pharmacological suppression of p38δ might represent such an approach. Conclusively, the p38δ-PKD pathway modulates both insulin secretion and β cell turnover and thus provides a unifying mechanism that integrates these two pathogenic features of human diabetes.

## Experimental Procedures

### Generation of Mice

The *p38δ* floxed mice were generated at Lexicon Pharmaceuticals (The Woodlands, TX) (for details, see the Supplemental Experimental Procedures). For generation of p38δ null mice, a targeting vector harboring LoxP sites within the 5′ UTR region and intron 1-2 (floxed exon 1) as well as a neomycin resistance cassette flanked by two FRT sites were electroporated into 129/SvEv^Brd^ (Lex-1) ES cells. Targeted ES cell clones were microinjected into C57BL/6 (albino) blastocysts to generate chimeric animals, which were bred to C57BL/6 (albino) females, and the resulting heterozygous offspring were bred with a protamine-Cre recombinase transgenic line to delete exon 1. Mice heterozygous for exon 1 deletion were backcrossed five times to the C57BL/6 background and intercrossed to generate homozygous knockout mice. All procedures involving animals were approved by the Veterinäramt des Kantons Zürich and conform to the relevant regulatory standards.

### Mouse Experiments

For the glucose tolerance test, 8-week-old male mice were fasted for 16 hr and then injected intraperitoneally (i.p.) with glucose (2 g/kg body weight [bw]). Glucose was measured with the Accu-Chek Aviva system from Roche. In experiments with the PI-PLC inhibitor U73122 (Sigma), mice were injected i.p. with U73122 (2.5 mg/kg bw) in DMSO or with DMSO 1 hr prior experiment. Insulin tolerance tests were performed on 8-week-old ad libitum-fed male mice injected i.p. with insulin (1 U/kg bw). Plasma insulin levels were measured with an insulin RIA kit (Linco). STZ (150 mg/kg bw) was injected once i.p., and blood glucose was measured each day. After 8 days, mice were sacrificed, serum was harvested, and the pancreas was isolated to measure insulin serum levels and insulin content, respectively. In STZ experiments with U73122, mice were injected i.p. with U73122 (2.5 mg/kg bw) in DMSO or with DMSO only, three times (day 1, 2, and 3) after STZ injection. Peripheral insulin resistance was induced by feeding of mice for 12 weeks with a high-fat diet (Research Diets, D12331). After this period, mice were subjected to insulin tolerance tests (1 U/kg bw) as well as glucose tolerance tests (1 g/kg bw).

### Islets Isolation and Insulin Secretion

Islets were isolated by collagenase perfusion (1.9 U/ml) of pancreas and subsequent digestion for 16 min at 37°C. Islets were handpicked and transferred to RPMI1640 media containing 5 mM glucose and maintained for 3 hr prior to the experiments or dispersed in calcium-free solution and then cultured as above. For static incubations, islets were subsequently preincubated for 60 min at 37°C in Krebs-Ringer bicarbonate buffer (pH 7.4), supplemented with 0.05% bovine serum albumin (BSA) and 2.8 mM glucose. After preincubation, the supernatant was discarded and the islets were incubated in buffer solution containing 2.8 or 16.7 mM glucose for 60 min at 37°C. For the rescue experiments, islets were incubated as described above but in presence of 10 μM of U73122 or 1 μM Gö6976 or DMSO. The islets were resuspended in acid ethanol and frozen for insulin content measurements. Insulin secretion data were expressed as percentage of pellet. Insulin secretion from INS1 and MIN6 cells was performed as described for islets, and data were normalized to nonstimulated control cells. Total insulin from pancreas was isolated by acid-ethanol extraction. Insulin content was normalized to the total weight of the pancreas.

### Cell Culture, Transfection and Cell Sorting

293T and MIN6 cells were maintained in Dulbecco's modified Eagle's medium (DMEM) (GIBCO, 31966) and INS1 in RPMI-1640 medium (Sigma R0883) according to standard procedures. Transfection of vectors, shRNA-mediated knockdown of p38δ, and siRNA against PKD1 in different cell lines is explained in detail in the Supplemental Experimental Procedures.

### Immunoprecipitation and Western Blotting

Transfected 293T were lysed in lysis buffer for 10 min at 4°C. After centrifugation at 13,000 rpm for 10 min, protein concentrations were measured in the lysate. One milligram of extract was incubated with the primary antibody (1:500) overnight at 4°C, followed by addition of 25 μl of Protein A/G Plus (Santa Cruz Biotechnology). Immobilized proteins were washed three times and analyzed by SDS-PAGE. For the experiment in Figure 3A, INS1 cells were stably transfected with a plasmid carrying *HA-p38δ^F324S^* or the relative empty control, lysed, and incubated with anti-HA antibody bound to agarose beads for 2 hr at 4°C. Immunoprecipitates were washed three times and analyzed by western blotting. Western blotting was performed according to standard procedures (for antibodies, see the Supplemental Experimental Procedures).

### In Vitro Kinase Assay

For the experiment in Figure 3B and Figure S5D, fully recombinant bacterial (Sigma) or Sf9 cell-derived (Cell Signaling Technology) GST-tagged PKD1, respectively, were subjected to an in vitro kinase assay with recombinant GST-tagged *p38δ*. For the experiment in Figure S5A, 293T cells were transfected with a plasmid carrying HA-tagged WT or constitutively active *p38δ* isoforms (D176A and F324S), lysed, and incubated with anti-HA antibody bound to agarose beads for 2 hr at 4°C. Immunoprecipitates were washed and incubated for 30 min at 30°C in kinase buffer containing 20 μM ATP, 5 μCi [γ-^32^P] ATP, and 2 μg recombinant HIS-tagged ATF-2 (Santa Cruz Biotechnology). For the experiment in Figure 3D and Figure S5C, 293T cells were transfected with human GFP-tagged PKD1-WT, PKD1-KD, or phospho mutant PKD1 expression plasmids and lysed in standard lysis buffer for 10 min at 4°C, followed by centrifugation. The cleared lysates were incubated overnight with α-GFP sepharose (Amersham), washed three times, and used as substrates for recombinant GST-p38δ in an in vitro kinase assay, as described above. For the experiment in Figure 3F, immunoprecipitated human GFP-tagged PKD1-WT, GPF-PKD1-KD, or phospho mutant GPF-PKD1 were equalized and used together with CREBtide as a substrate in an in vitro kinase assay. Kinase activity was measured by autoradiography caused by incorporation of phosphate into the CREBtide peptide (KRREILSRRPSYR) at 30°C with a final concentration of 50 μM [γ-^32^P] ATP.

### Immunofluorescence

Immunofluorescence (IF) for insulin (Linco) and glucagon (Linco) was performed on paraformaldehyde-fixed pancreatic sections. Relative islet area was measured as percentage of insulin-positive staining sections. Ten different sections per mouse were used. IF for giantin (Covance), anti-furin convertase (Abcam), and GM130 (BD Biosciences) were performed on methanol-acetone fixed INS1 and MIN6 cells and primary β cells. Islets were dispersed in single cells as described in single-cell capacitance measurements, incubated in RPMI 1640 medium containing 5 mM glucose for 2 hr in the presence or absence of the PI-PLC inhibitor U73122 (10 μM), the PKC inhibitor Gö6976 (1 μM), or DMSO, and then spun onto poly-lysine-coated slides (700 G for 5 min).

### TUNEL Assay

For the detection of apoptosis, TUNEL (terminal deoxynucleotide transferase-mediated dUTP nick end labeling) staining was performed on paraformaldehyde-fixed pancreatic sections according to the manufacturer's instruction (Roche). Apoptotic cells were quantified in islets area within the section. Three independent sections per mouse were used.

### Real-Time RT-PCR

RNA was extracted with TRIzol (Invitrogen). cDNA was synthesized with the Ready-To-Go You-Prime First-Strand beads (Amersham). Primers are indicated in the Supplemental Experimental Procedures.

### LC-MS/MS

A detailed description of the protein digest, peptide analysis with liquid chromatography-tandem mass spectrometry, and database searches is given in the Supplemental Experimental Procedures.

### Electrophysiology and Measurement of [Ca^2+^]_i_

Whole-cell currents and exocytosis were recorded and analyzed with EPC-9 patch-clamp amplifiers and the software Pulse+Pulsefit (Heka Electronic). [Ca^2+^]_i_ was assessed in freshly isolated islets with a dual wavelength PTI system (PTI, Monmouth, NJ) fitted to an inverted microscope (for details, see the Supplemental Experimental Procedures).

### Statistical Analyses

Statistical significance was calculated with an ANOVA with post hoc Tukey's test and student's unpaired t test. Significance was accepted at the level of p < 0.05. For electron microscopy, statistical analyses are indicated in the Supplemental Experimental Procedures.

## Supplemental Data

Document S1. Supplemental Experimental Procedures, 15 Figures, and Two Tables
