# Supplementary material for: Regulation of PKD by the MAPK p38δ in Insulin Secretion and Glucose Homeostasis
Source: Cell. 2009 Jan 23;136(2):235–48. doi: 10.1016/j.cell.2008.11.018 (PMC2638021; doi:10.1016/j.cell.2008.11.018)
Supplement: Document S1. Supplemental Experimental Procedures, 15 Figures, and Two Tables [file mmc1.pdf]

Cell, Volume *136*

Supplemental Data

Regulation of PKD by the MAPK p38δ in Insulin Secretion and Glucose Homeostasis

Grzegorz Sumara, Ivan Formentini, Stephan Collins, Izabela Sumara, Renata Musialek, Bernd Bodenmiller, Reshma Ramracheya, Dorothée Caille, Huiping Jiang, Kenneth A. Platt, Paolo Meda, Rudolf Aebersold, Patrik Rorsman, and Romeo Ricci

Supplemental Experimental Procedures

Generation of mice. The *p38δ* floxed mice were generated at Lexicon Pharmaceuticals (The Woodlands, TX). The *p38δ* targeting vector was derived using the Lambda KOS system (Wattler et al., 1999). The Lambda KOS phage library, arrayed into 96 superpools, was screened by PCR using primers *p38δ*-2 [5'-CCTCGCAGGACCGCCACCAC-3'] and *p38δ*-4 [5'-GGGCGCCAGGTAGGTCTTGG-3']. The PCR-positive phage superpools were plated and screened by filter hybridization using the 114 bp amplicon derived from primers *p38δ*-2 and *p38δ*-4 as a probe. Three pKOS genomic clones, pKOS-50, pKOS-63, and pKOS-70 were isolated from the library screen and confirmed by sequence and restriction analysis. Gene-specific arms (5'-GAACGTACCTGGGCGAGGCGGCAGGT-3') and (5'-AGTTACAGCCTTGGAGAACCAGATTCG-3') were appended by PCR to a yeast selection cassette containing the URA3 marker. The yeast selection cassette and pKOS-50 were co-transformed into yeast, and clones that had undergone homologous recombination to replace a 346 bp region containing exon 1 with the yeast selection cassette were isolated. This 346 bp fragment was independently amplified by PCR and cloned into the intermediate vector pLF-Neo introducing flanking LoxP sites and a Neo selection cassette (*p38δ*-pLFNeo). The yeast cassette was subsequently replaced with the *p38δ*-pLFNeo selection cassette to complete the conditional *p38δ* targeting vector that has exon 1 flanked by LoxP sites. The Not I linearized targeting vector was electroporated into 129/SvEv^{Brd} (Lex-1) ES cells. G418/FIAU resistant ES cell clones were isolated, and correctly targeted clones were identified and confirmed by Southern analysis using a 413 bp 5' external probe (57/58), generated by PCR using primers *p38δ*-57 [5'-GGCACATGGCAAGCACGTATG-3'] and *p38δ*-58 [5'- CTAATTCTGAGTATAGATCTTTGC-3'], and a 313 bp 3' internal probe (60/59), amplified by PCR using primers *p38δ*-60 [5'-CGTGGGTGCTAAGGGTTGAAC-3'] and *p38δ*-59 [5'-GGTAGCTGGAAGGCAGGAGTG-3']. Southern analysis using probe 57/58 detected a 5.5 Kb wild type band and 3.2 Kb mutant band in Eco RI + Sfi I digested genomic DNA while probe 60/59 detected a 15.5 Kb wild type band and 13.8 Kb mutant band in Xho I + Sfi I digested genomic DNA. Targeted ES cell clones were microinjected into C57BL/6 (albino) blastocysts to generate chimeric animals which were bred to C57BL/6 (albino) females, and the resulting heterozygous offspring were bred with a protamine-Cre recombinase transgenic line (O'Gorman et al., 1997) to delete exon 1 (Figure S1). Mice heterozygous for exon 1 deletion were backcrossed

five times to the C57BL/6 background and intercrossed to generate homozygous knockout mice.

Cell culture, transfection and cell sorting. Transfection of 293T cells was performed using calcium phosphate precipitation. INS1 cells were transfected with the pcDNA3.1-HA-*p38δ*, pcDNA3.1-HA-*p38δ*^{F324S} expression plasmid or the empty vector using Fugene reagent (Roche) and selected with 500 µg/ml neomycin (Sigma). Knockdown of *p38δ* in MIN6 was obtained by lentivirus-mediated transduction of short hairpin against *p38δ* RNA (sequences are provided in supplemental experimental procedures) and selection with puromycin (4 µg/ml). Transient knockdown of PKD1 in INS1 or MIN6 cells was performed by electroporation. Briefly, cells were resuspended in OPTImem (Invitrogen) and electroporated at 270 V and 960 µF with 2 µM of siRNA duplex using the Gene Pulser Xcell (Bio-Rad). Forty-eight hours after electroporation, cells were subjected to analysis as described below. Transfection of INS1 cells with GFP or GFP-tagged PKD WT or mutant forms was achieved by electroporation as described above. To enrich for GFP-positive cells, INS1 cells were sorted 24 hours after transfection using FACS Aria Cell Sorter (BD) equipped with an argon laser emission of 488 nm. Non-transfected cells were used as a control to set the cutoff value for background fluorescence.

Antibodies used for Western blotting. The following antibodies were used: rabbit-anti-p38δ (Santa Cruz Biotechnology, Figure 1A, 4A) and sheep-anti-p38δ (generous gift of Dr. Ana Cuenda, Figure 6B, S8, S12A, S15A), anti-phospho-PKD (Ser744/748 and Ser916) (Cell Signaling Technology), anti-PKD1, anti-HIS, anti-hemagglutinin (HA), anti-Tubulin and anti-Actin (all from Sigma).

LC-MS/MS analysis. The samples were analyzed on a hybrid LTQ-FTICR mass spectrometer (Thermo, San Jose, CA) interfaced with a nanoelectrospray ion source. Chromatographic separation of peptides was achieved on an Eksigent nano LC system (Eksigent Technologies, Dublin, CA, USA), equipped with a 11 cm fused silica emitter, 75 µm inner diameter (BGB Analytik, Böckten, Switzerland), packed in-house with a Magic C18 AQ 5 µm resin (Michrom BioResources, Auburn, CA, USA). Peptides were loaded from a cooled (4°C) Spark Holland auto sampler and separated using ACN/water solvent system containing 0.1 % formic acid with a flow rate of 300 nl/min. Peptide mixtures were separated with a gradient from 3 to 30 % ACN in 90 min. Up to three data-dependent MS2 spectra were acquired in the linear ion trap for each FT-MS spectral acquisition range, the latter acquired at 100,000 FWHM nominal resolution settings with an overall cycle time of approximately 1 s. Charge state screening was employed to select for ions with at least two charges and rejecting ions with undetermined charge state. For each peptide sample, a standard data-dependent acquisition method on the three most intense ions per MS-scan was used and a threshold of 200 ion counts was used for triggering an MS2 attempt.

LC-MS/MS data analysis. The MS2 data were, dependant on the sample analyzed, searched against the human (v3.23) and mouse IPI database (v3.26) non redundant database using SORCERER-SEQUEST(TM) (Eng et al., 1994) v3.0.3, which was run on the SageN Sorcerer2 (Thermo Electron, San Jose, CA, USA). For the in silico digest, trypsin was defined as protease, cleaving after K and R (if followed by P the cleavage was not allowed). Two missed cleavages and one non-tryptic terminus were

allowed for the peptides that had a maximum mass of 6000 Da. The precursor ion tolerance was set to 25 p.p.m. The data were searched allowing phosphorylation (+79.9663 Da) of serine, threonine and tyrosine as a variable modification and carboxyamidomethylation of cysteine (+57.0214 Da) residues as a fixed modification. In the end, the search results obtained bySequest were subjected to statistical filtering using PeptideProphet (Keller et al., 2002) (v3.0) and ProteinProphet (v3.0) (Keller et al., 2005). The phosphopeptide tandem mass spectra were furthermore manually inspected and tested for the presence of a neutral loss peak of – 98 Da which is indicative for a phosphopeptides under CID (Bodenmiller et al., 2007). Finally, also the MS1 mass shift between the non-phosphopeptide and phosphopeptide of +79.9663 Da was used to verify the results.

Electrophysiology. Exocytosis, expressed as an increase in capacitance was elicited by either a train of 10 500-ms depolarisations delivered at 1 Hz, from -70 mV to 10 mV or by infusion of a Ca^{2+}/EGTA-buffer with an intracellular free $[Ca^{2+}]_i$ of 1.5 μM. The electrodes were pulled from borosilicate glass and had a resistance of 4 to 7 MΩ. When filled with the electrode solution, the access resistance was <15 MΩ and seal resistance >1 GΩ. β-cells were identified as previously described (Gopel et al., 2004). In the Ca^{2+} dialysis experiments, the intracellular solution consisted of (in mM) 125 CsCl, 10NaCl, 1 $MgCl_2$, 10 EGTA, 9 $CaCl_2$, 3 Mg-ATP, 0.1 cAMP, 5 Hepes (pH 7.15). When exocytosis was triggered by depolarization, the intracellular solution contained (in mM) 125 glutamate, 10 CsCl, 10 NaCl, 1 $MgCl_2$, 5 Hepes, 0.05 EGTA, 0.1 cAMP and 3 MgATP (pH 7.15). For all experiments, cells were superfused with a solution containing 118 NaCl, 20 TEA-Cl, 5.6 KCl, 2.6 $CaCl_2$, 1.2 $MgCl_2$, 25 Hepes, 5 glucose (pH 7.4). The temperature was kept at +32 to +34°C.

Measurement of $[Ca^{2+}]_i$. Islets were loaded with 3 μM fura-2AM in the presence of 0.007% w/v pluronic acid (Invitrogen) for 25 min at 37ºC. Islets were held in the chamber using a fire polished borosilicate glass pipette. In solution composed of (in mM) 140 NaCl, 3.6 KCl, 2 $NaHCO_3$, 0.5 NaH_2PO_4, 0.5 $MgSO_4$, 5 Hepes (pH 7.4 with NaOH), 2.6 $CaCl_2$ preheated with temperature controller (TC 324B, Warner instrument corporation) at 37ºC. Glucose and KCl were included in the extracellular medium as indicated. When KCl was elevated, NaCl was correspondingly reduced. The fluorofore was excited at 350 and 380 nm. Emitted light was collected at 510 nm. Ratiometric measurements were done at 25 Hz and calibrated as described (Grynkiewicz et al., 1985). R_{max} was measured after addition of ionomycin (conc: ~1 μM) in the presence of 10 mM $CaCl_2$ at the end of the experiment. R_{min} was established by replacing the extracellular solution with 0 mM $CaCl_2$. Background was obtained after quenching fura-2 fluorescence with 1 mM $MnCl_2$.

Transmission electron microscopy (TEM). To evaluate the type, abundance and distribution of secretory granules, sections of $p38\delta^{+/+}$ and $p38\delta^{\Delta/\Delta}$ mice were prepared for conventional electron microscopy. Quantitative analyses of pancreatic β cells have been assessed by TEM as previously described (Stefan et al., 1987). Briefly, the areas of cytoplasm, Golgi apparatus and peripheral membrane compartment (defined as a band of 300 nm thickness, about the diameter of one secretory granule, along the cell membrane) were evaluated by semi-automatic planimetry of photographs taken at a magnification of 19000x, using a graphic tablet and the Leica Qwin software (Leica, Glattbrugg, Switzerland). The areas of secretory granules in these 3 compartments and the length of cell membrane were similarly evaluated. The number of mature

(small very electron dense core, large electron lucent halo) and immature granules (medium dense core, thin peripheral halo) was further scored in all compartments. From these data, the volume density of secretory ganules was calculated in each β-cell by dividing the cumulated areas of these organelles by that of the cytoplasm, Golgi or membrane compartment, respectively. The numerical density of granules apposed to the cell membrane was given by dividing the number of granules scored in the membrane compartment by the length of the cell membrane. The proportion of mature and immature granules in each compartment was given by dividing the number of each granule type by the total number of granules. Data were expressed as mean ± SEM and compared by either analysis of variance and ad Scheffe's ad hoc t-test (volume and numerical density of granules) or by the Chi-square test (distribution of secretory granules), as provided by the SPSS software (SPSS, Chicago, USA).

Primers and shRNA sequences.

Name	Sequence
p38δ shRNA forward	5'-GATCCCCGATGCTGGAGCTGGATGTGTTC AAGAGACACATCCAGCTCCAGCATCTTTTTA-3
p38δ shRNA revers	5'-AGCTTAAAAAGATGCTGGAGCTGGATGTG TCTCTTGAACACATCCAGCTCCAGCATCGGG-3'
p38δ RT-PCR forward	5'-ATGAGCCTCACTCGGAAAAGG-3'
p38δ RT-PCR reverse	5'-GCATGTGCTTCAAG AGCAGAA-3'
18s RT-PCR forward	5'-GTTCCGACCATAAACGATGCC-3'
18s RT-PCR reverse	5'-TGGTGGTGCCCTTCCGTCAAT-3'
BI5-12	5'-GCTCAGCTTCTTGATGGCCAC-3'
PGK-162r	5'-GGATGTGGAATGTGTGCGAGG-3'
Delta-1	5'-GAGCTACCCAAGACCTACCTG-3'
Delta-2	5'-ACGTACCTGGGCGAGGCGGCA-3'
r-PKD1 siRNA seq1	5'- AAACAGGAAGAGATGTAGCTATT-3'
m(r)-PKD1 siRNA seq2	5'- AAAGTCAGCTTCGTAATGAGGTT-3'

Supplemental References

Bodenmiller, B., Mueller, L.N., Mueller, M., Domon, B., and Aebersold, R. (2007). Reproducible isolation of distinct, overlapping segments of the phosphoproteome. Nat Methods *4*, 231-237.

Eng, J.K., McCormack, A.L., and Yates, J.R. (1994). An approach to correlate tandem mass spectral data of peptides with amino acid sequences in a protein database. Journal Of The American Society For Mass Spectrometry *5*, 976-989.

Gopel, S., Zhang, Q., Eliasson, L., Ma, X.S., Galvanovskis, J., Kanno, T., Salehi, A., and Rorsman, P. (2004). Capacitance measurements of exocytosis in mouse pancreatic alpha-, beta- and delta-cells within intact islets of Langerhans. J Physiol *556*, 711-726.

Grynkiewicz, G., Poenie, M., and Tsien, R.Y. (1985). A new generation of Ca2+ indicators with greatly improved fluorescence properties. J Biol Chem *260*, 3440-3450.

Keller, A., Eng, J., Zhang, N., Li, X.J., and Aebersold, R. (2005). A uniform proteomics MS/MS analysis platform utilizing open XML file formats. Mol Syst Biol *1*, 2005 0017.

Keller, A., Nesvizhskii, A.I., Kolker, E., and Aebersold, R. (2002). Empirical statistical model to estimate the accuracy of peptide identifications made by MS/MS and database search. Anal Chem *74*, 5383-5392.

O'Gorman, S., Dagenais, N.A., Qian, M., and Marchuk, Y. (1997). Protamine-Cre recombinase transgenes efficiently recombine target sequences in the male germ line of mice, but not in embryonic stem cells. Proc Natl Acad Sci U S A *94*, 14602-14607.

Stefan, Y., Meda, P., Neufeld, M., and Orci, L. (1987). Stimulation of insulin secretion reveals heterogeneity of pancreatic B cells in vivo. J Clin Invest *80*, 175-183.

Wattler, S., Kelly, M., and Nehls, M. (1999). Construction of gene targeting vectors from lambda KOS genomic libraries. BioTechniques *26*, 1150-1156, 1158, 1160.

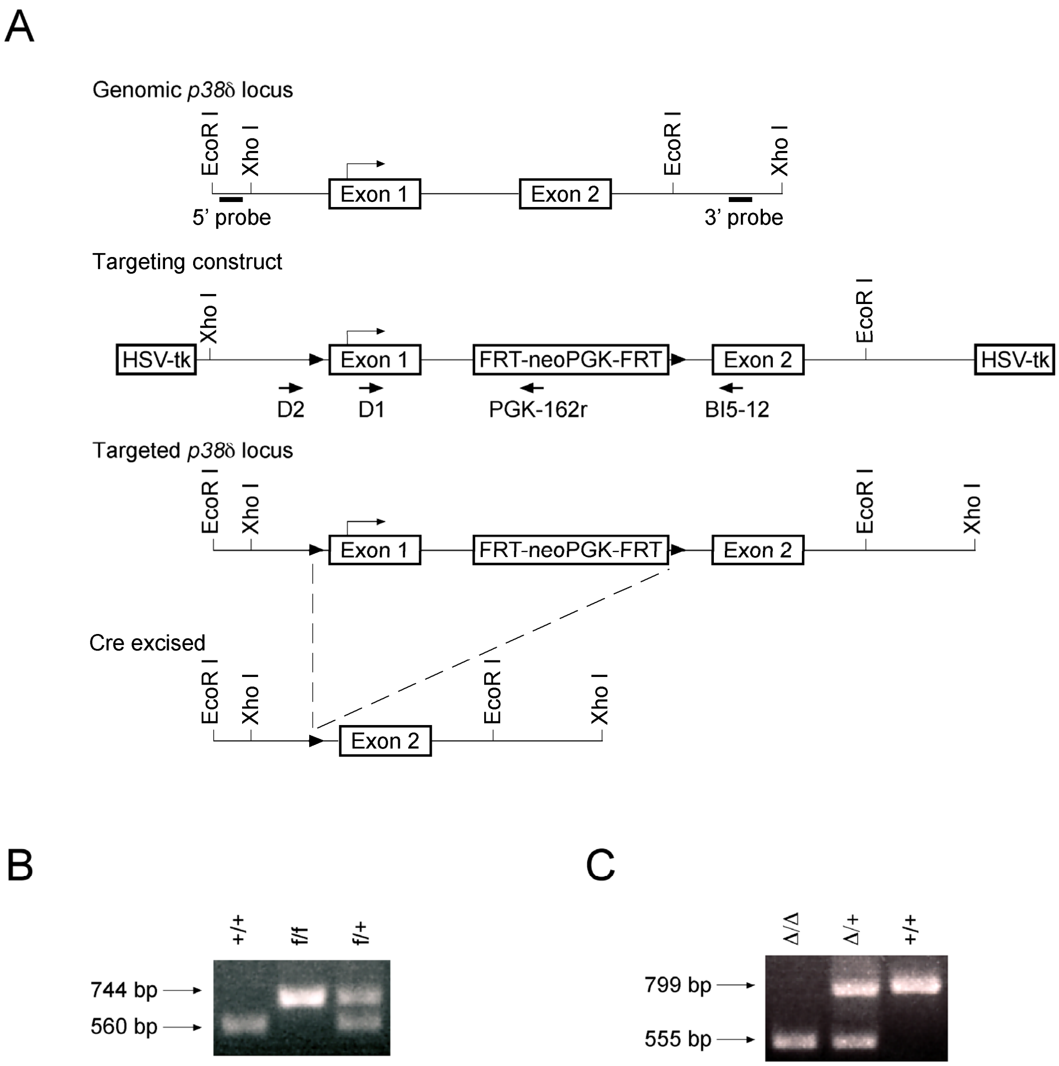

Figure S1. Generation of *p38δ* floxed mice and *p38δΔ/Δ* mice using Protamine Cre. (A) Schematic representation of the targeting strategy employed to generate a floxed allele of *p38δ*. Exons 1 and 2 of the *p38δ* open reading frame are represented by rectangles, thin lines represent untranslated regions of the *p38δ* locus. The neomycin resistance gene (for positive selection) flanked by two FRT sites (FRT-neoPGK-FRT) and the HSV-tk cassettes (for negative selection) are indicated. The arrows indicate the position of the primers for genotyping, loxP sites are shown as triangles. The strategy for generation of the targeted allele is described in SOM. **(B)** PCR analysis of genomic DNA isolated from adult *p38δ*+/+, *p38δf/*+ and *p38δf/f* mice using D1, PGK-162r and BI5-12 primers, yielded a 744 bp band corresponding to the floxed allele and a 560 bp band corresponding to the wild type allele **(C)** PCR analysis of genomic DNA isolated from mice carrying deletion of *p38δ* by Protamine Cre; *p38δ*Δ/Δ, *p38δ*Δ/+ and *p38δ*+/+. PCR was performed using D2 and BI5-12 and yielded a 799 bp band corresponding to the wild type allele and a 555 bp band corresponding to the deleted *p38δ* allele.

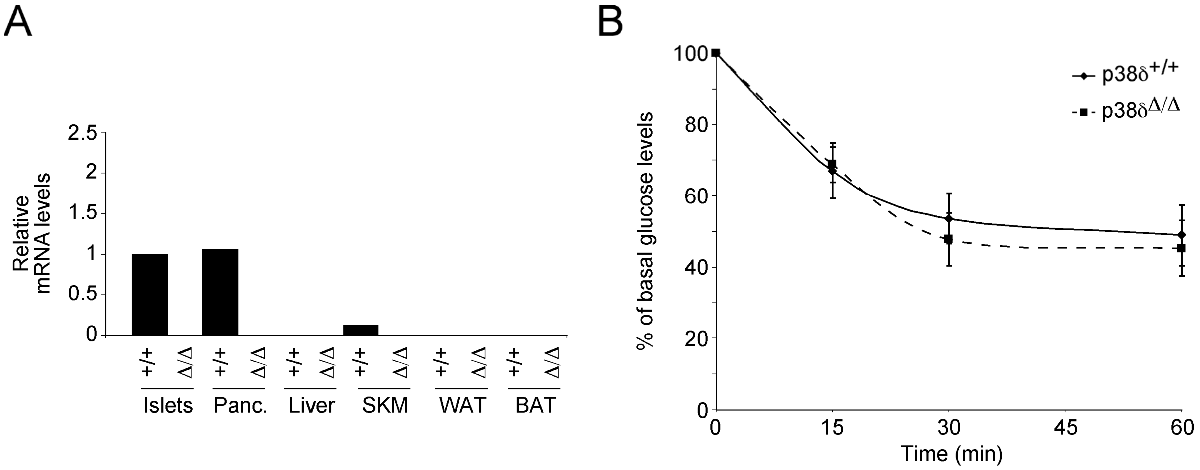

Figure S2. Abundant expression of *p38δ* in pancreas and normal insulin sensitivity in *p38δΔ/Δ* mice. (A) Relative expression of *p38δ* mRNA, as tested by quantitative real time (RT)-PCR, showed expression of *p38δ* in isolated islets and total pancreas (Panc.) of *p38δ+/+* but not *p38δΔ/Δ* mice, and a low expression in skeletal muscle (SKM) but not in liver, white (WAT) and brown adipose tissue (BAT). Expression levels were normalized to those of *18S* rRNA. (**B**) Insulin tolerance tests. Insulin (1 U/kg) was injected intraperitoneally in ad libitum fed mice and glucose was measured at indicated time points. Insulin sensitivity was equal in *p38δΔ/Δ* (squares and dotted line, n=5) and *p38δ+/+* (diamonds and solid line, n=6) mice. All error bars indicate ±SEM.

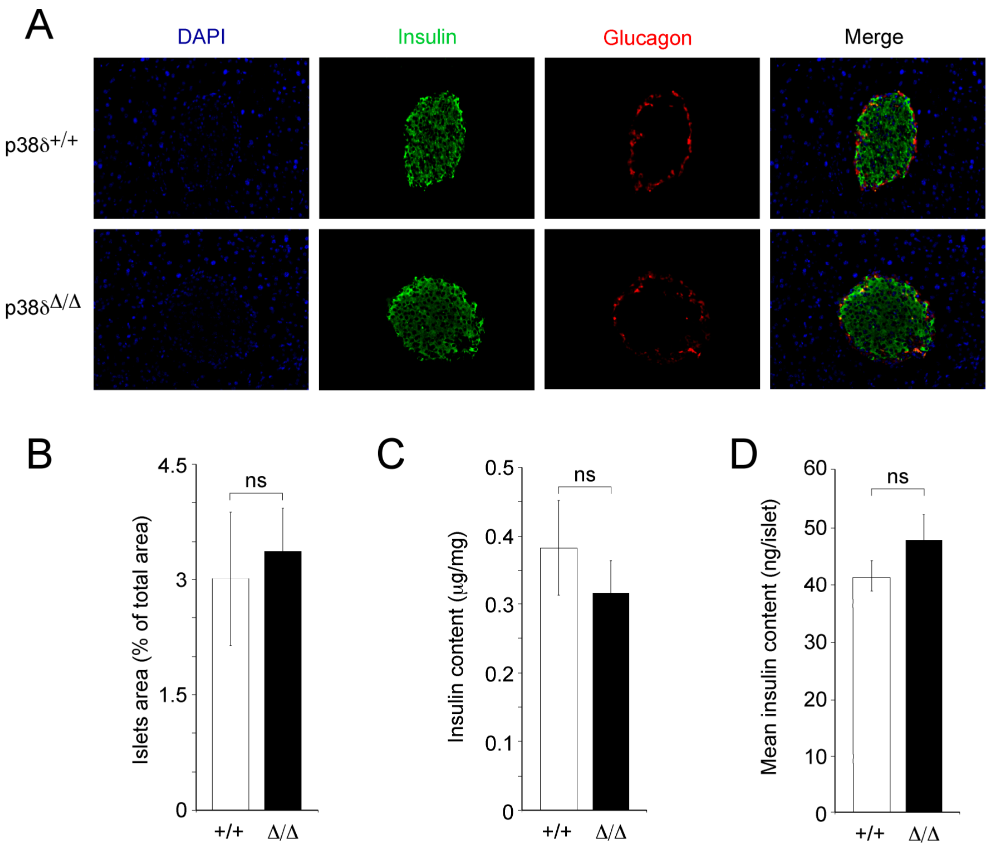

Figure S3. Unaltered islet architecture, islet area and insulin content in p38δΔ/Δ mice. (A) Insulin (middle left panel, green) and glucagon (middle right panel, red) immunolabelling revealed comparable islet characteristics in *p38δΔ/Δ* (lower panels) and *p38δ+/+* (upper

panel) mice. DAPI was used to stain nuclei (left panel). Merged pictures are provided in the right panels. **(B)** Quantitative histological assessment of total islet area in relation to total pancreatic area. No significant differences in *p38*δΔ/Δ (black bars, n=3) compared to *p38*δ+/+ (white bars, n=3) could be observed. **(C)** Total insulin extracted from pancreas measured by radio-immuno assay. No significant difference in total insulin content (μg/mg) was detected between *p38*δΔ/Δ (black bars, n=3) and *p38*δ+/+ mice (white bars, n=3). **(D)** The mean insulin content per isolated islet was also similar in *p38*δΔ/Δ (black bars, n=14) and *p38*δ+/+ mice (white bars, n=16). All error bars indicate ±SEM.

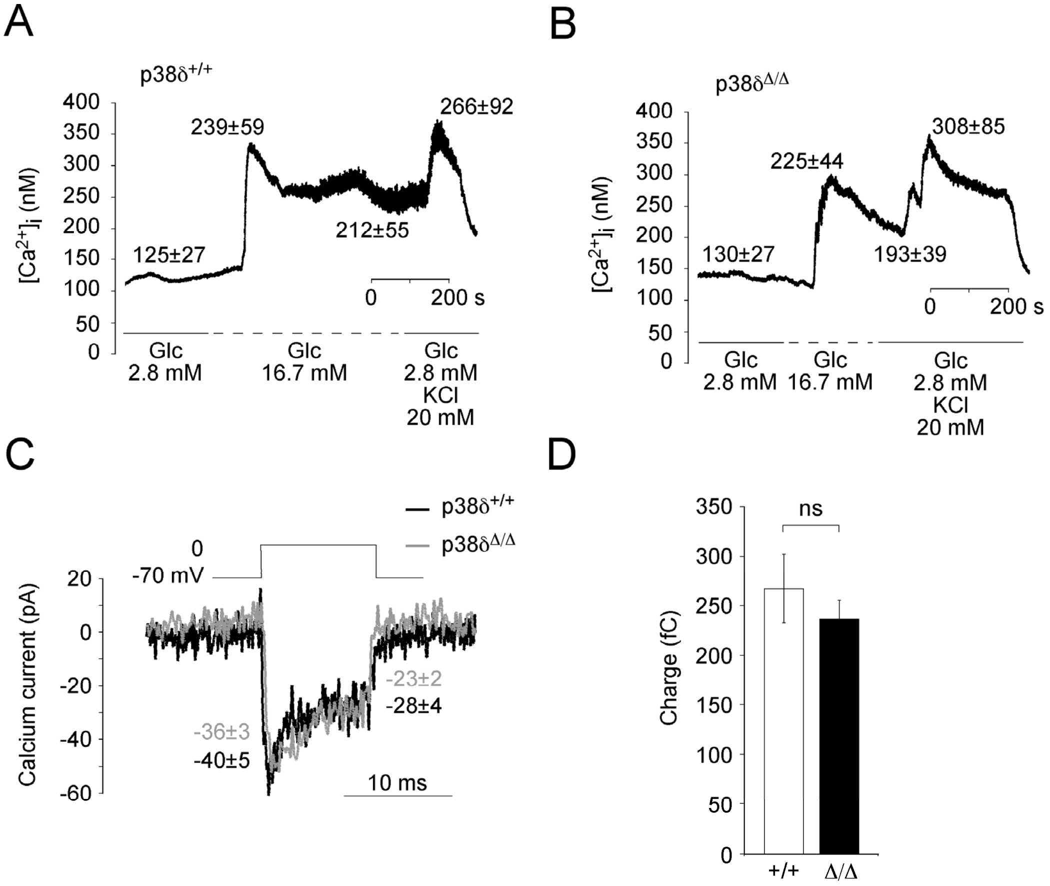

Figure S4. No effect of *p38δ* deletion on [Ca2+]i handling and voltage-gated Ca2+ currents. **(A)** and **(B)** Representative traces of intracellular calcium ([Ca2+]i) in pancreatic islets of wild type (*p38*δ+/+) (n=16) **(A)** and *p38*δ null (*p38*δΔ/Δ) mice (n=12) **(B)** under basal conditions and after stimulation with 16.7 mM Glucose and 20 mM KCl as indicated. Average responses ±SEM are indicated for basal [Ca2+]i, peak glucose response, plateau glucose response, and the peak response to KCl. No significant difference could be observed between *p38*δ+/+ and *p38*δΔ/Δ islets. **(C)** Representative Ca2+ current in isolated pancreatic islets of *p38*δ+/+ (black solid line, n=20) and *p38*δΔ/Δ (grey solid line, n=17) mice evoked by a depolarisation from -70 mV to 0 mV. Currents are shown together with respective means ±SEM of the peak and sustained currents (measured at the end of the pulse). No significant differences was observed between *p38*δ+/+ and *p38*δΔ/Δ islets. **(D)** Summary of the average charge entry (derived from the area under the curve during the depolarisation) during the 10-ms pulse from -70 mV to 0 mV in *p38*δ+/+ (+/+) and *p38*δΔ/Δ (Δ/Δ) β cells. No significant difference was observed between *p38*δ+/+ and *p38*δΔ/Δ islets. All error bars indicate ±SEM.

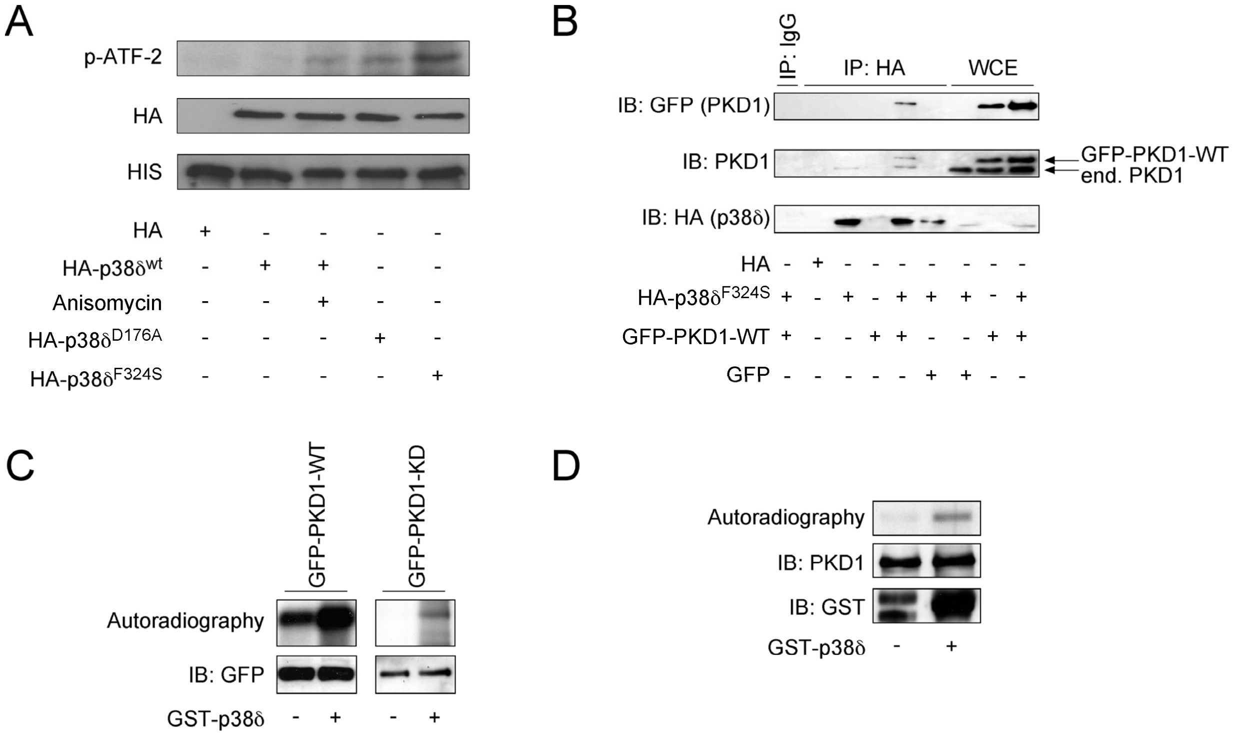

Figure S5. p38δ interacts with PKD1 and phosphorylates it in vitro. (A) In vitro kinase assay using recombinant histidin (HIS)-tagged ATF-2 as a substrate of immunoprecipitated ectopic hemagglutinin (HA)-tagged p38δ. 293T cells were transfected with expression vectors containing HA (lane 1), wild type HA-p38δ (HA-p38δwt) without (lane 2) and with anisomycin (a known activator of p38δ) (lane 3), constitutive active HA-p38δD176A (lane 4) and constitutive active HA-p38δF324S (lane 5). After in vitro kinase reaction, proteins were separated by SDS-PAGE. In vitro kinase activity was tested by measuring phosphorylation of ATF-2 (p-ATF-2) (upper lane). Equal loading of p38δ and ATF-2 was confirmed using an HA antibody and a HIS antibody, respectively (middle and lower lanes). Amino acid substitution F324S more efficiently enhanced p38δ activity compared to D176A. **(B)** Western blot with indicated immunoprecipitates (IP) and whole cell extracts (WCE) from 293T cells transfected with indicated constructs confirms physical interaction with exogenous (GFP-PKD1-WT) and endogenous PKD1 (end. PKD1). **(C)** *In vitro* kinase assay with and without recombinant p38δ and immunoprecipitated GFP-tagged wild type (GFPPKD1-WT) and kinase-dead PKD1 (GFP-PKD1-KD. **(D)** *In vitro* kinase assay with and without recombinant p38δ and Sf9 cell-derived GST-tagged PKD1.

A

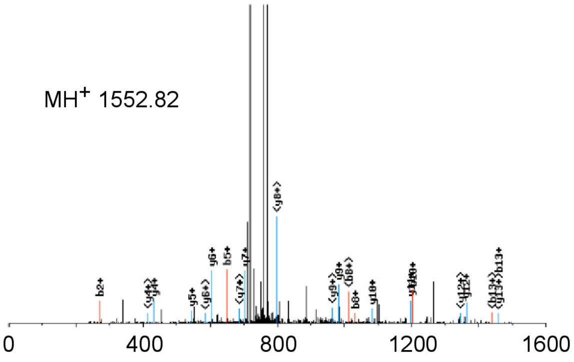

…^{201}RLpSNVpSLTGLGTVR^{214}…

b^+	#	AA	#	y^+	y^{2+}
157.1089	1	R	14		
270.1930	2	L	13	1476.6864	738.8471
437.1914	3	S	12	1363.6024	682.3051
551.2343	4	N	11	1196.6040	598.8059
650.3027	5	V	10	1082.5611	541.7845
817.3011	6	S	9	983.4927	492.2503
930.3851	7	L	8	816.4943	408.7511
1031.4328	8	T	7	703.4103	352.2090
1088.4543	9	G	6	602.3626	301.6852
1201.5383	10	L	5	545.3411	273.1745
1258.5598	11	G	4	432.2571	216.6324
1359.6075	12	T	3	375.2356	188.1217
1458.6759	13	V	2	274.1879	137.5979
	14	R	1	175.1195	88.0637

B

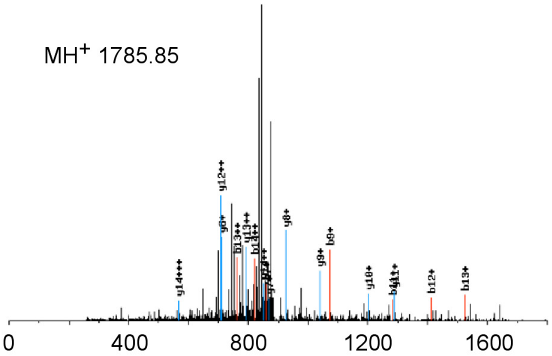

…^{253}SNpSQSYIGRPIQLDK^{267}…

b^+	b^{2+}	#	AA	#	y^+	y^{2+}	y^{3+}
88.0399	44.5238	1	S	15			
202.0828	101.5453	2	N	14	1698.8216	849.9147	566.9458
369.0811	185.0445	3	S	13	1584.7787	792.8933	528.9314
497.1397	249.0738	4	Q	12	1417.7803	709.3941	473.2653
584.1717	292.5898	5	S	11	1289.7218	645.3648	430.5791
747.2351	374.1214	6	Y	10	1202.6897	601.8488	401.5685
860.3191	430.6635	7	I	9	1039.6264	520.3171	347.2140
917.3406	459.1742	8	G	8	926.5423	463.7751	309.5193
1073.4417	537.2248	9	R	7	869.5209	435.2643	290.5122
1170.4945	585.7511	10	P	6	713.4198	357.2138	238.4785
1283.5785	642.2932	11	I	5	616.3670	308.6874	206.1275
1411.6371	706.3225	12	Q	4	503.2829	252.1454	168.4329
1524.7212	762.8645	13	L	3	375.2244	188.1161	125.7467
1639.7481	820.3780	14	D	2	262.1403	131.5741	88.0520
		15	K	1	147.1134	74.0606	49.7097

C

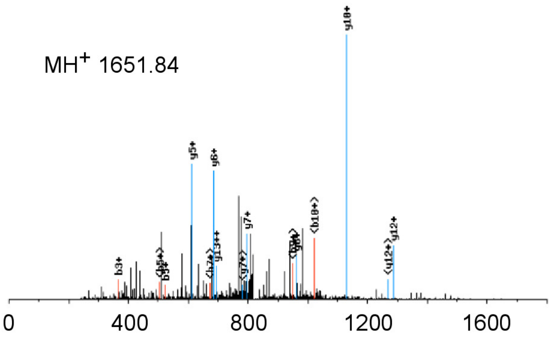

…^{748}pSVVGTPAYLAPEVLR^{762}…

b^+	#	AA	#	y^+	y^{2+}
168.0062	1	S	15		
267.0746	2	V	14	1484.8477	742.9278
366.1430	3	V	13	1385.7793	693.3935
423.1645	4	G	12	1286.7109	643.8593
524.2122	5	T	11	1229.6894	615.3486
621.2649	6	P	10	1128.6417	564.8248
692.3020	7	A	9	1031.5890	516.2984
855.3654	8	Y	8	960.5518	480.7798
968.4494	9	L	7	797.4885	399.2482
1039.4865	10	A	6	684.4044	342.7061
1136.5393	11	P	5	613.3673	307.1876
1265.5819	12	E	4	516.3146	258.6612
1364.6503	13	V	3	387.2720	194.1399
1477.7344	14	L	2	288.2036	144.6057
	15	R	1	175.1195	88.0637

Figure S6. LC-MS/MS analysis of digested GST-PKD1 fusion protein. 293T cells transfected with GST-tagged PKD1 were lysed and incubated with Glutathione-epharose.The precipitates were eluted and subjected to an *in vitro* kinase assay. The tandem mass spectra of the 201-214 **(A)**, 253-267 **(B)** and 748-762 **(C)** Phosphopeptides as well as the annotated peptide ion fragments are shown. In red the b ions and in blue the y ions are shown. Each of the spectra displays an intense neutral loss fragment ion of -98 Da indicating the presence of a phosphorylated amino acid.

A

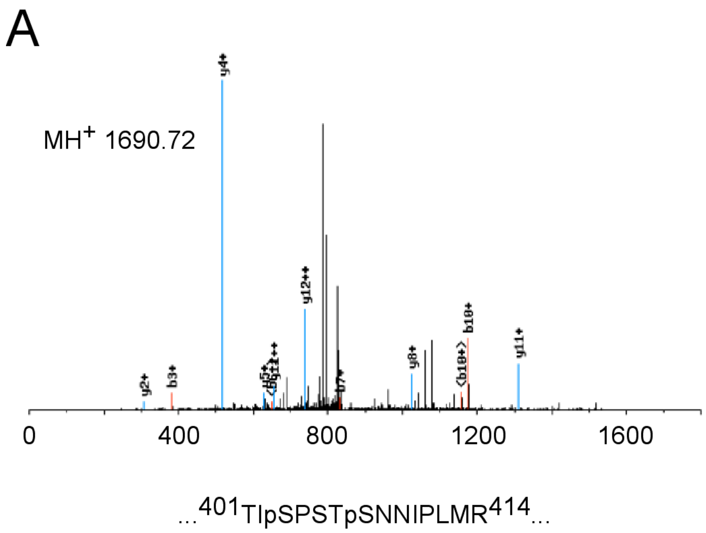

B

b^+	#	AA	#	y^+	y^{2+}
102.0555	1	T	14		
215.1396	2	I	13	1589.6800	795.3439
382.1379	3	S	12	1476.5959	738.8019
479.1907	4	P	11	1309.5976	655.3027
566.2227	5	S	10	1212.5448	606.7763
667.2704	6	T	9	1125.5128	563.2603
834.2688	7	S	8	1024.4651	512.7365
948.3117	8	N	7	857.4667	429.2373
1062.3546	9	N	6	743.4238	372.2158
1175.4387	10	I	5	629.3809	315.1944
1272.4914	11	P	4	516.2968	258.6523
1385.5755	12	L	3	419.2440	210.1259
1516.6160	13	M	2	306.1600	153.5839
	14	R	1	175.1195	88.0637

Figure S7. p38δ directly phosphorylates PKD1 in two specific conserved serine residues. 293T cells transfected with GST-tagged PKD1 were lysed and incubated with Glutathione-sepharose.The precipitates were eluted and subjected to an *in vitro* kinase assay. **(A)** The tandem mass spectrum of the 401-414 phosphopeptide is shown. **(B)** Annotated peptide ion fragments. In red the b ions and in blue the y ions are shown. The spectrum displays an intense neutral loss fragment ion of -98 Da indicating the presence of a phosphorylated amino acid.

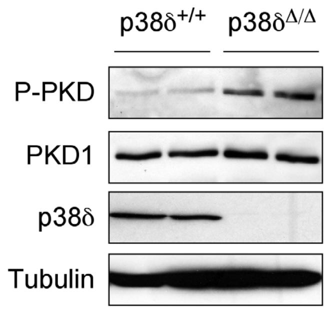

Figure S8. Loss of *p38δ* leads to increased activation of PKD in pancreas. Western blotting with total cell lysates from pancreas of *p38δ+/+* and p38δΔ/Δ mice revealed an enhanced autophosphorylation of PKD (Serine 916). Tubulin was used to confirm equal loading.

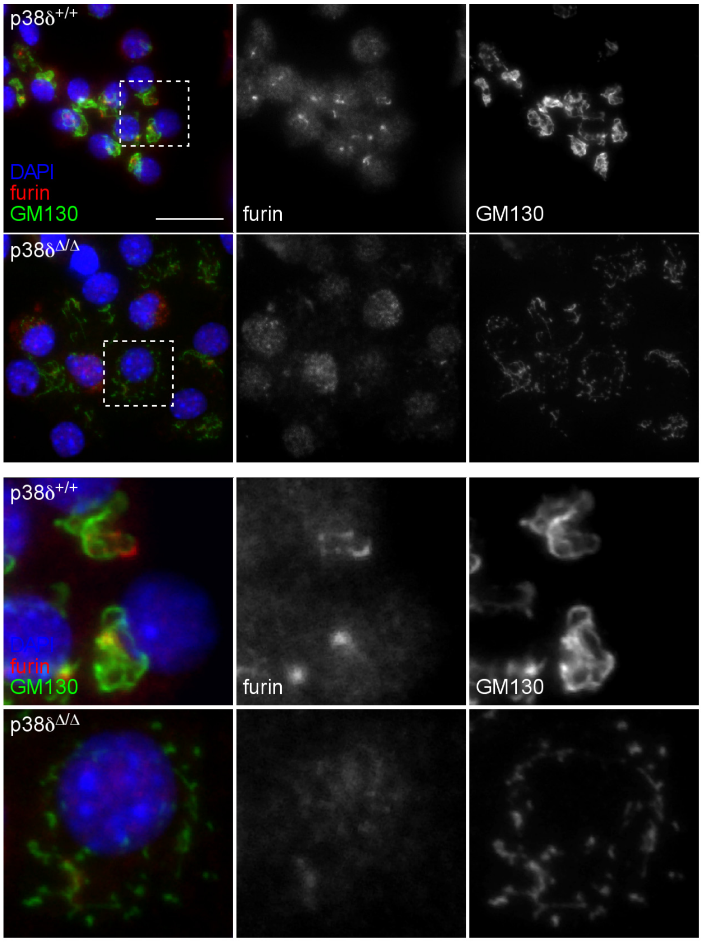

Figure S9. *p38*δ deletion leads to dispersion of furin convertase and GM130 in primary pancreatic β cells. *p38*δ-deficient (*p38*δΔ/Δ) and wild type (*p38*δ+/+) pancreatic β cells were analyzed by immunofluorescence microscopy using antibody against Golgi-proteins furin convertase (furin, red) and GM130 (green). DNA was counterstained with DAPI (blue). Boxes in the left column outline the areas that have been magnified in the panels below. Size bar 10 µm.

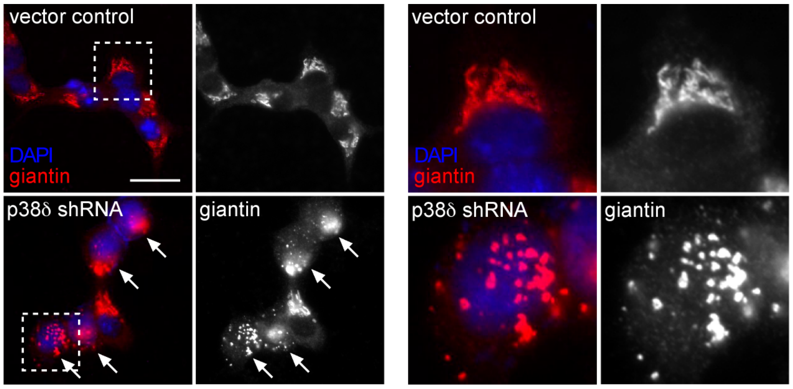

Figure S10. *p38δ* deletion leads to dispersion of giantin in MIN6 cells. MIN6 cells stably expressing shRNA against *p38*δ (*p38*δ shRNA) and cells expressing the empty vector (vector control) were cultured and analyzed by immunofluorescence microscopy using antibodies against giantin (red). DNA was stained with DAPI (blue). Note that *p38*δ shRNA expressing cells show dislocalization of giantin (arrows). Size bar 10 µm.

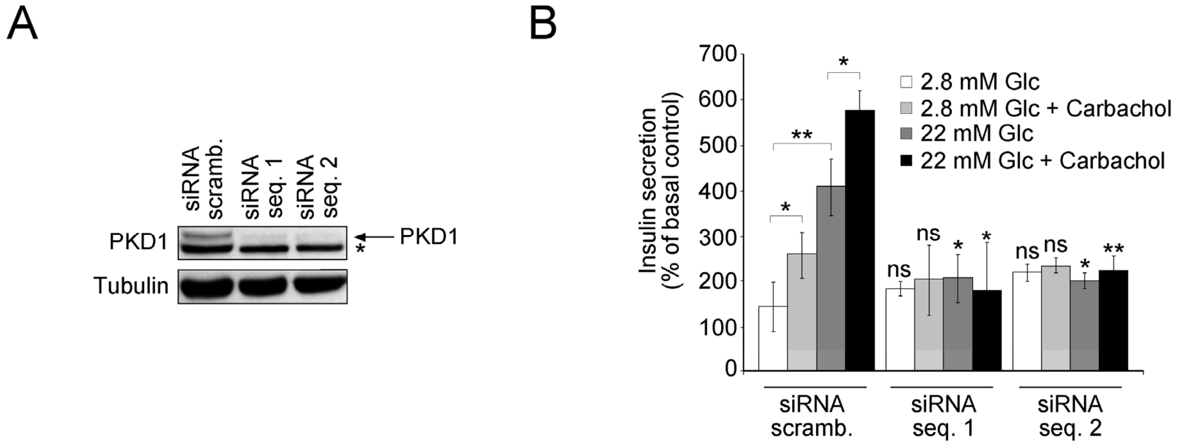

Figure S11. Inactivation of *PKD1* in INS-1 cells inhibits stimulated insulin secretion. (A) Western blot (WB) with total cell lysates from INS-1 cells transfected with scrambled control siRNA (lane 1, siRNA scramb.), siRNA with sequence 1 (lane 2, siRNA seq. 1) and siRNA with sequence 2 (lane 3, siRNA seq. 2). Knockdown was about 80% for both sequences (*unspecific band). Tubulin WB indicated equal loading (lower panel). **(B)** Insulin secretion was measured in INS-1 cells transfected with indicated siRNAs under basal and stimulatory glucose (Glc) with and without carbachol (1μM) as indicated. Both stimuli enhanced insulin secretion (*p<0.05 and **p<0.01). Inactivation of *PKD1* reduced glucose- and carbacholstimulated insulin secretion (*p<0.05 and **p<0.01). Basal insulin secretion was not affected by these genetic perturbations (ns, not significant). All error bars indicate ±SEM.

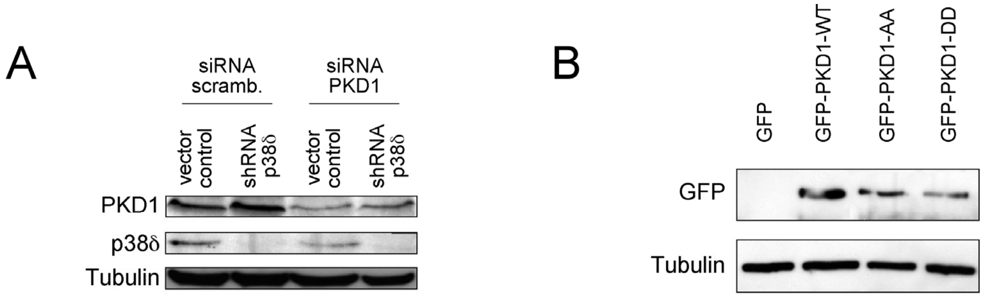

Figure S12. Inactivation of PKD1 and p38δ in MIN6 cells and stable expression of PKD1 mutants in INS1 cells. (A) Western blot with total cell lysates from MIN6 cells stably expressing shRNA against p38δ (shRNA p38δ) or a control vector (vector control) transfected with scrambled control siRNA (siRNA scramb.) or siRNA against PKD1 (siRNA PKD1). Knockdown by siRNA was about 60%. Tubulin WB indicated equal loading (lower panel). **(B)** Western blot with total cell lysates from INS1 cells overexpressing GFP or GFP-tagged WT and mutant forms of PKD1. GFP positive cells were enriched by FACS sorting with a purity of 95% before conducting experiments (data not shown). Tubulin WB indicated equal loading (lower panel).

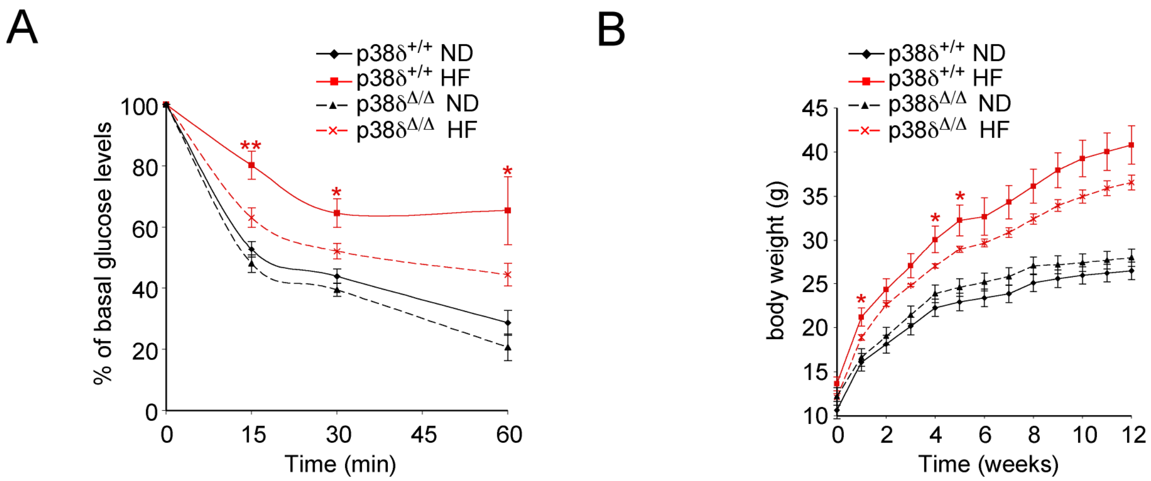

Figure S13. Reduced weight gain and attenuated insulin resistance in *p38δΔ/Δ* compared to *p38δ*+/+ mice on a high-fat diet. (A) Insulin tolerance tests. Insulin (1 U/kg) was injected intraperitoneally in ad libitum fed mice and glucose was measured at indicated time points. Insulin sensitivity was equal in *p38*δΔ/Δ (squares and dotted line, n=7) and *p38*δ+/+ (diamonds and solid line, n=5) mice on a normal diet. However, insulin sensitivity was significantly improved in *p38*δΔ/Δ (n=11) compared to *p38*δ+/+ mice (n=6) on a HF diet (*p<0.05 and **p<0.01). **(B)** Body weights of *p38*δΔ/Δ and *p38*δ+/+ mice on a high-fat (HF, n=11 and n=6, respectively) or normal diet (ND, n=7 and n=5, respectively) were determined at indicated time points. *p38*δΔ/Δ mice gained less weight on a HF diet compared to *p38*δ+/+ (*p<0.05), while no differences could be observed on a normal diet. All error bars indicate ±SEM.

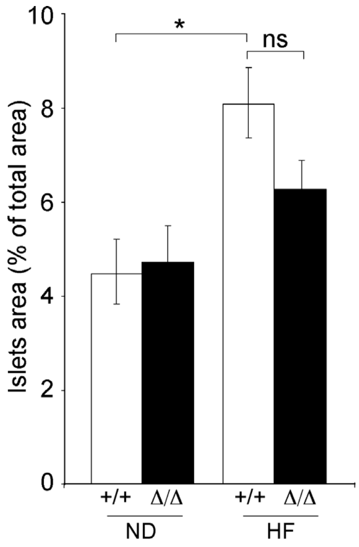

Figure S14. Islet growth under high-fat feeding conditions is similar in *p38δΔ/Δ* and *p38δ*+/+ mice. Quantitative histological assessment of total islet area in relation to total pancreatic area. Islet growth in response to high-fat feeding (HF) in *p38*δ+/+ (+/+, n=3) mice was significantly enhanced compared to mice on a normal diet (ND). The islet area of p38δΔ/Δ (Δ/Δ, n=3) mice on a high-fat diet was also increasing compared to mice on a normal diet but differences were not significant. However, no signifcant (ns) difference in the islet area between *p38*δ+/+ and *p38*δΔ/Δ mice on a high-fat diet could be observed. All error bars indicate ±SEM.

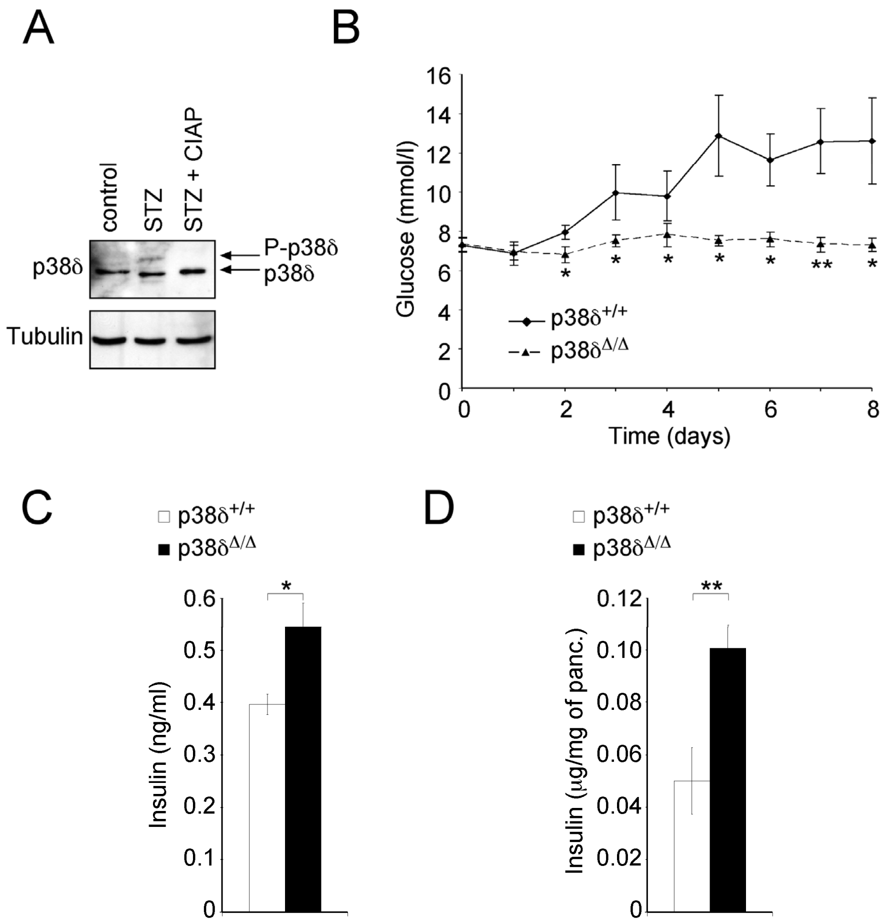

Figure S15. *p38δΔ/Δ* mice are protected against streptozotocin-induced pancreatic β cell failure. (A) SDS-PAGE with polyacrylamide-bound Mn2+-Phos-tag to measure the activity of p38δ in starved INS-1 cells in response to streptozotocin (STZ). Phosphorylation of p38δ upon STZ was abolished by CIAP phosphatase treatment.**(B)** Blood glucose levels in *p38*δΔ/Δ mice (triangles and dotted line, n=8) and *p38*δ+/+ mice (diamonds and solid line, n=8) in response to streptozotocin (STZ) at indicated time points (*p<0.05, **p<0.01). **(C)** Blood insulin levels in *p38*δΔ/Δ mice (black bar, n=8) and *p38*δ+/+ mice (white bar, n=8) 8 days after STZ treatment (*p<0.05). **(D)** Total pancreatic insulin content in *p38*δΔ/Δ mice (black bar, n=8) and *p38*δ+/+ mice (white bar, n=8) 8 days after STZ treatment (**p<0.01). All error bars indicate ±SEM.

Table S1. Ultrastructural evaluation of secretory granules in primary pancreatic β-cells.

genotype	# mice / islets / cells scored	Volume fractions of secretory granules (%)			membrane-apposed granules	% dense core / pale granules		
		Cyto-plasm	Golgi	Membrane	(# /µm)	cytoplasm	Golgi	Mem-brane
p38δ$^{+/+}$	3 / 6 / 90	20 ± 1.1	4.1 ± 0.5	75 ± 4.0	6.61 ± 0.22	92 / 8	60 / 40	98 / 2
		t =0.41 p=0.73	*t=0.21 p=0.23*	*t =0.77 p=0.46*	*t=0.95 p= 0.34*	*X^2=4.21 p=0.88 dense core granules*	*X^2=3.32 p=0.81 pale*	
p38δ$^{\Delta/\Delta}$	3 / 6 / 107	19 ± 3.2	5.2 ± 0.7	80 ± 5.5	7.01 ± 0.34	90 / 10	54 / 46	95 / 5

Values are mean ± SEM of the indicated number of β-cells.

Table S2. Putative proteins interacting with HA-p38δ^{F324S} revealed by LC-MS/MS.

No.	Protein/Gene name[(a)]	Number of peptides[(b)]	Coverage (% of the mass)	Accessions[(c)]
1	Protein kinase D1 (Prkcm)	36	48.8	Q15139 NP_002733
2	Elongation factor G 1	8	13.3	Q96RP9 NP_079272
3	Elongation factor 1-α 1	10	34.8	P68104 NP_001393
4	Isoform M2 of Pyruvate kinase isozymes M1\M2	18	36.9	P14618 NP_002645
5	ATP synthase subunit alpha	8	21.7	P25705 NP_001001937
6	Fructose-bisphosphate aldolase A	7	26.1	P04075 NP_000025
7	L-lactate dehydrogenase B chain	10	22.5	P07195 NP_002291
8	14-3-3 protein zeta\delta	5	27.5	P63104 NP_003397
9	Glyceraldehyde-3-phosphate dehydrogenase	7	37.2	P04406 NP_002037
10	GTP-binding nuclear protein Ran	5	29.2	P62826 NP_006316
11	Phosphoglycerate mutase 1	6	31.1	P18669 NP_002620
12	Rab GDP dissociation inhibitor beta	6	18.4	P50395 NP_001485
13	40S ribosomal protein S3	7	41.2	P23396 NP_000996
14	Isoform 3 of Heterogeneous nuclear ribonucleoprotein K	9	28.5	P61978 NP_002131
15	Histone H1.2	10	25.8	P16403 NP_005310
16	Ras GTPase-activating protein-binding protein 1	6	20.8	Q13283 NP_005745
17	ATP-dependent RNA helicase DDX3X	13	36.9	O00571 NP_001347
18	ATP-dependent RNA helicase A	10	10.7	Q08211 NP_001348
19	Poly [ADP-ribose] polymerase 1	8	12.2	P09874 NP_001609
20	ATP-dependent RNA helicase DDX1	5	9.1	Q92499 NP_004930
21	Isoform Short of RNA-binding protein FUS	34	37.7	P35637 NP_004951
22	Isoform Long of Splicing	28	34.1	P23246

	factor, proline- and glutamine-rich			NP_005057
23	Isoform Long of TATA-binding protein-associated factor 2N	13	21.6	Q92804 NP_631961
24	Non-POU domain-containing octamer-binding protein	14	21.1	Q15233 NP_031389
25	Eukaryotic translation initiation factor 5B	5	6.2	O60841 NP_056988
26	HSPA5 protein	8	18.2	P11021 NP_005338
27	Histone H1.4	8	25.1	P10412 NP_005312
28	Interleukin enhancer-binding factor 3	5	11.5	Q12906 NP_036350
29	HNRPA1 protein	6	14.2	P09651 NP_112420
30	Isoform 1 of Plasminogen activator inhibitor 1 RNA-binding protein	9	27.0	Q8NC51 NP_001018077
31	Polyadenylate-binding protein 1	11	21.9	P11940 NP_002559
32	Malate dehydrogenase, mitochondrial precursor	7	29.0	P40926 NP_005909
33	Elongation factor 2	9	13.4	P13639 NP_001952
34	Nucleosome assembly protein 1-like 4	5	24.8	Q99733 NP_005960
35	Neutral alpha-glucosidase AB precursor	7	13.9	Q14697 NP_938148
36	Phosphoglycerate kinase 1	6	19.1	P00558 NP_000282
37	D-3-phosphoglycerate dehydrogenase	5	13.9	O43175 NP_006614
38	40S ribosomal protein S14	6	42.4	P62263 NP_001020241
39	Ubiquitin-activating enzyme E1	12	18.1	P22314 NP_003325
40	T-complex protein 1 subunit zeta	7	18.8	P40227 NP_001753
41	Endoplasmin precursor	10	23.8	P14625 NP_003290
42	Fatty acid synthase	9	5.3	P49327 NP_004095
43	Inorganic pyrophosphatase	6	28.7	Q15181 NP_066952
44	Profilin-1	6	48.6	P07737

				NP_005013
45	Myristoylated alanine-rich C-kinase substrate	11	47.9	P29966 NP_002347
46	Creatine kinase B-type	11	34.9	P12277 NP_001814
47	plastin 3	7	15.6	P13797 NP_005023
48	T-complex protein 1 subunit theta	10	22.4	P50990 NP_006576
49	glutamyl-prolyl tRNA synthetase	5	5.3	P07814 NP_004437
50	Adenosylhomocysteinase	6	15.3	P23526 NP_000678
51	Thioredoxin-like protein 2	6	14.7	P49321 NP_002473
52	Heterogeneous nuclear ribonucleoprotein	6	25.8	P61978 NP_112552
53	T-complex protein 1 subunit epsilon	7	21.6	P48643 NP_036205
54	Carbonyl reductase [NADPH] 1	10	42.2	P16152 NP_001748
55	Elongation factor 1-δ	5	28.4	P29692 NP_001951
56	Calnexin precursor	13	21.1	P27824 NP_001019820
57	Peroxiredoxin-6	5	28.6	P30041 NP_004896
58	L-lactate dehydrogenase A chain	7	25.0	P00338 NP_005557
59	Ras GTPase-activating-like protein IQGAP1	6	15.4	P46940 NP_003861
60	ATP-dependent DNA helicase 2 subunit 1	6	10.7	P12956 NP_001460
61	T-complex protein 1 subunit delta	8	27.6	P50991 NP_006421
62	Poly [ADP-ribose] polymerase 1	10	13.6	P09874 NP_001609
63	Nuclear migration protein nudC	6	21.5	Q9Y266 NP_006591
64	T-complex protein 1 subunit gamma	8	19.3	P49368 NP_005989
65	T-complex protein 1 subunit beta	10	25.6	P78371 NP_006422
66	Multifunctional protein ADE2	5	13.4	P22234 NP_001072992
67	Stress-induced-phosphoprotein 1	7	14.9	P31948 NP_006810
68	Non-POU domain-	9	21.2	Q15233

	containing octamer-binding protein			NP_031389
69	Isoform 1 of Protein disulfide-isomerase A6 precursor	7	25.5	Q15084 NP_005733
70	Clathrin heavy chain 1	10	9.0	Q00610 NP_004850
71	Calcyclin-binding protein	5	47.0	Q9HB71 NP_055227
72	ADP-ribosylation factor 3	5	38.1	P61204 NP_001650
73	Transgelin-2	6	40.2	P37802 NP_003555
74	Alpha-enolase	7	18.2	P06733 NP_001419
75	60S acidic ribosomal protein P2	6	69.6	P05387 NP_000995
76	Elongation factor 1-beta	15	44.9	P24534 NP_001032752
77	14-3-3 protein beta\alpha	7	26.6	P31946 NP_003395
78	Eukaryotic initiation factor 4A-I	8	29.6	P60842 NP_001407
79	Protein disulfide-isomerase A3	9	26.3	P30101 NP_005304
80	Nucleolin	11	19.4	P19338 NP_005372
81	Nuclease sensitive element-binding protein 1	5	35.5	P67809 NP_004550
82	ATP synthase subunit alpha	8	21.7	P25705 NP_001001937
83	Protein TFG	8	16.4	Q92734 NP_001007566
84	Lamina-associated polypeptide 2 isoform alpha	5	11.2	P42166 NP_003267
85	Insulin-like growth factor 2 mRNA binding protein 2	5	12.2	Q9Y6M1 NP_001007226

[a]Validated protein identifications was made on the basis of Protein Prophet score of 1.0.
[b]Number of unique different peptides for each protein entry. A Peptide Prophet score of 0.5 was used.
[c] SwissProt entry and RefSeq collection number.
